# Molecularly Imprinted Ratiometric Fluorescent Sensors for Analysis of Pharmaceuticals and Biomarkers

**DOI:** 10.3390/s24217068

**Published:** 2024-11-02

**Authors:** Jingyi Yan, Siwu Liu, Dani Sun, Siyuan Peng, Yongfei Ming, Abbas Ostovan, Zhihua Song, Jinmao You, Jinhua Li, Huaying Fan

**Affiliations:** 1 School of Pharmacy, Key Laboratory of Molecular Pharmacology and Drug Evaluation (Yantai University), Ministry of Education, Collaborative Innovation Center of Advanced Drug Delivery System and Biotech Drugs in Universities of Shandong, Yantai University, 32 Qingquan Road of Laishan District, Yantai 264005, China; yjy1091897645@163.com (J.Y.); liujiu645@126.com (S.L.); zhihuasong08@yeah.net (Z.S.); 2 Coastal Zone Ecological Environmental Monitoring Technology and Equipment Shandong Engineering Research Center, Shandong Key Laboratory of Coastal Environmental Processes, Yantai Institute of Coastal Zone Research, Chinese Academy of Sciences, 17 Chunhui Road of Laishan District, Yantai 264003, China; sun19961212@163.com (D.S.); a.ostovan@yic.ac.cn (A.O.); 3 School of Life Science, Ludong University, Yantai 264025, China; 17658128539@163.com (S.P.); mingyf@163.com (Y.M.); 4 College of Chemistry and Chemical Engineering, Shaoxing University, Shaoxing 312000, China; jmyou6304@163.com

**Keywords:** molecularly imprinted polymers, ratiometric fluorescent sensor, point-of-care testing, pharmaceuticals, biomarkers, review

## Abstract

Currently, analyzing pharmaceuticals and biomarkers is crucial for ensuring medication safety and protecting life and health, and there is an urgent need to develop new and efficient analytical techniques in view of the limitations of traditional analytical methods. Molecularly imprinted ratiometric fluorescent (MI-RFL) sensors have received increasing attention in the field of analytical detection due to their high selectivity, sensitivity and anti-interference ability, short response time, and visualization. This review summarizes the recent advances of MI-RFL sensors in the field of pharmaceuticals and biomarkers detection. Firstly, the fluorescence sources and working mechanisms of MI-RFL sensors are briefly introduced. On this basis, new techniques and strategies for preparing molecularly imprinted polymers, such as dummy template imprinting, nanoimprinting, multi-template imprinting, and stimulus-responsive imprinting strategies, are presented. Then, dual- and triple-emission types of fluorescent sensors are introduced. Subsequently, specific applications of MI-RFL sensors in pharmaceutical analysis and biomarkers detection are highlighted. In addition, innovative applications of MI-RFL sensors in point-of-care testing are discussed in-depth. Finally, the challenges of MI-RFL sensors for analysis of pharmaceuticals and biomarkers are proposed, and the research outlook and development trends of MI-RFL sensors are prospected.

## 1. Introduction

Nowadays, the analysis of pharmaceuticals and biomarkers is increasingly becoming the focus of scientific research and public health. Pharmaceutical analysis includes drug quality control, drug metabolism analysis, and drug residues analysis to ensure the safety and efficacy of pharmaceuticals. The emergence of drug residues as a global public health challenge is of particular concern [1]. Residues of veterinary drugs, pesticides, and other chemical substances not only pollute the natural environment and disrupt the ecological balance but also accumulate through the food chain, posing a long-term threat to human health, such as inducing chronic diseases and immune system disorders [2]. Biomarkers are biochemical indicators that reflect changes in the structure or function of body systems and organs [3]. Pharmaceuticals and biomarkers are closely related [4]. The metabolic process of pharmaceuticals in the body produces various biomarkers, and changes in these biomarkers can reflect the efficacy and safety of pharmaceuticals. At the same time, biomarkers can also be used as diagnostic indicators of diseases and provide a basis for pharmaceutical development and treatment. Given the importance of pharmaceuticals and biomarkers analysis, it is quite imperative to find efficient, accurate, rapid, and practical analytical methods.

Currently, the commonly used analytical technologies of high-performance liquid chromatography (HPLC) [5], capillary electrophoresis (CE) [6], gas chromatography-mass spectrometry (GC-MS) [7], LC-MS/MS [8], and enzyme-linked immunosorbent assay (ELISA) [9] encounter limitations, such as complex operation, expensive equipment, and long analysis time [10]. Point-of-care testing (POCT) is an emerging rapid detection method that allows rapid detection at the sampling points using a portable analytical instrument. Its significant advantage is that it is easy to operate without complex pretreatment steps, thus reducing the dependence on professional operators and greatly reducing the risk of deterioration, contamination, or loss of samples that may occur during transportation and storage, further improving the accuracy and reliability of test results [11,12].

In recent years, fluorescence sensors have received increasing attention as an effective tool for efficient and rapid detection [13,14,15]. Especially, ratiometric fluorescent sensors are becoming research hotspots [15,16,17,18] since they have target-independent internal self-correction ability and can provide more accurate analysis results and visualization by eliminating external disturbances often perplexing the conventional single-emission systems [13,14], such as background fluorescence and environmental changes. For example, Miao et al. [18] constructed a three-emission ratio fluorescence sensor based on carbon point-gold nanocluster nanocomposites for the detection of tetracycline. Ratio fluorescence assays have been widely used in the analysis of pharmaceuticals and biomarkers [17,18,19,20]. Meanwhile, molecularly imprinted polymers (MIPs) prepared by molecular imprinting technology (MIT) also have been increasingly utilized for the selective recognization and adsorption of target compounds (e.g., pharmaceuticals and biomarkers) in complex matrices [21,22]. By incorporating MIPs into ratiometric fluorescence sensors, the constructed molecularly imprinted ratiometric fluorescent (MI-RFL) sensors can combine the advantages of MIPs recognization and ratiometric fluorescence detection and thereby possess superiorities, such as excellent selectivity, high sensitivity, fast response, high accuracy and reliability, simple operation, and easy visualization in detection, even POCT of trace analytes from complex matrices [20,23,24]. 

**Figure 1:**
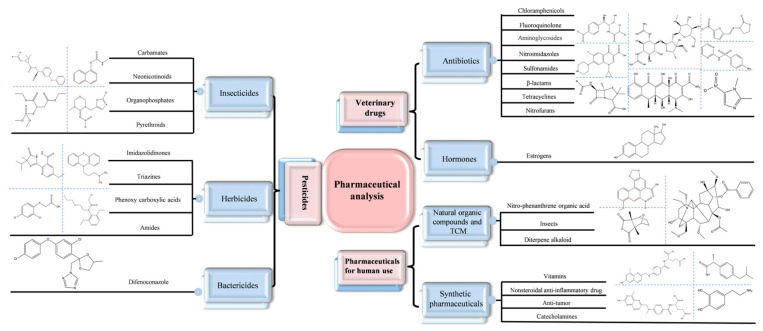
Classification of pharmaceuticals involved in the MI-RFL sensing analysis and representative substances.

MI-RFL sensors usually adopt dual- or triple-emission design according to the number of emission peaks, and post-imprinting mixing (PIMix) and post-imprinting modification (PIMod) strategies are increasingly employed for constructing the sensor to improve the overall analytical performance [25]. The sensors have displayed versatile applications, especially in the analysis of pharmaceuticals and biomarkers. Figure 1 illustrates the classification of pharmaceuticals involved in recent years and typical representative substances. For example, Arslan et al. [26] constructed a MI-RFL sensor based on MIPs-coated quantum dots (MIP-QDs) to selectively detect double-stranded DNA (dsDNA). Chen et al. [27] developed a kind of bimetallic nanozyme triple-emission MI-RFL sensor for the ultrasensitive analysis of triclosan (TCS). In addition, with the rapid development of POCT technology, the MI-RFL sensors show great potential in practical applications. By combining test strips, smartphones, microfluidic paper chips, and other portable devices, it can realize rapid and accurate on-site detection of drug residues and biomarkers and provide efficient and convenient solutions for many fields, such as agriculture, animal husbandry, food safety, and environmental monitoring.

**Figure 2:**
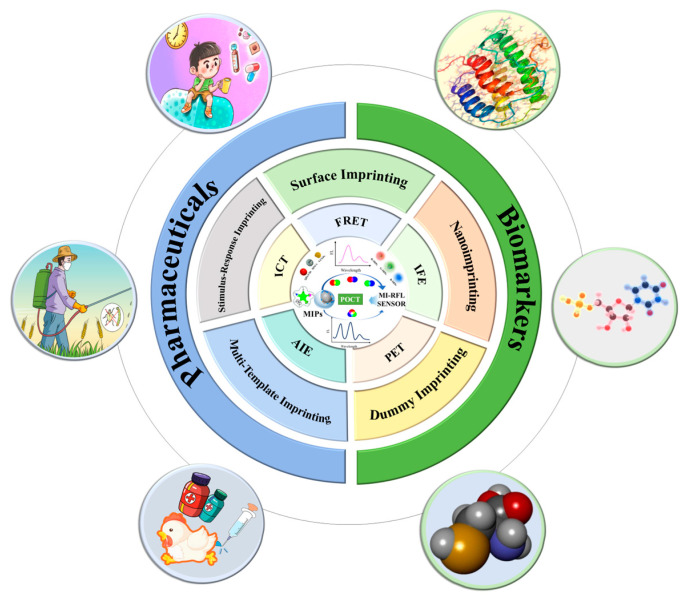
The main content of this review.

Therefore, this work reviews recent research advances in MI-RFL sensors for analysis of pharmaceuticals and biomarkers since 2019. Figure 2 schematically shows the main content of the review. Firstly, the fluorescence sources of MI-RFL sensors, their working mechanisms, and new techniques and strategies for preparing MIPs are overviewed. Then the dual- and triple-emission types of fluorescent sensors are introduced. Typical applications of MI-RFL sensors for pharmaceutical analysis and biomarkers detection are summarized. This is followed by an in-depth discussion of innovative applications of MI-RFL sensors in POCT, including emerging technologies, such as test strips, smartphones, and microfluidic chips. Finally, the multiple challenges of MI-RFL sensors in pharmaceuticals and biomarkers analysis, such as material selection, preparation process, sensitivity, and stability, are proposed, and the research future of MI-RFL sensors is also prospected, focusing on the integration of new materials and technologies, as well as green and smart strategies for more efficient, environmentally friendly, and sustainable development.

## 2. Fluorescent Signal Source and Working Mechanism of MI-RFL Sensor

### 2.1. Fluorescent Signal Source

The MI-RFL sensor generates two or more emission peaks through its fluorescence signal source at a specific excitation wavelength, resulting in a fluorescence response signal and a reference signal [28,29]. Therefore, in the construction of an MI-RFL sensor, the crucial point is to select the appropriate fluorescence source, according to the change of fluorescence signal after the target is identified, to achieve high sensitivity and selective quantitative detection of the target. Currently, fluorescent materials commonly used to construct MI-RFL sensors mainly include fluorescent dyes, QDs, carbon dots (CDs), upconversion nanoparticles (UCNPs), and metal nanoclusters (MNCs) [30].

#### 2.1.1. Fluorescent Dyes

Fluorescent dyes (also known as fluorophores/reactive dyes) can be described simply as molecules (essentially non-proteins) that perform their function under a microscope by absorbing light of a given wavelength and re-emitting it to a longer wavelength. Rhodamine and fluorescein are some of the most common fluorescent dyes; these fluorescent dyes are relatively inexpensive and easy to handle and can be excited efficiently by a wide range of excitation wavelengths, making them very attractive for these applications. They are characterized by narrow (slightly structured) absorption and emission bands that tend to map onto each other [30,31]. Chu et al. [32] synthesized an MI-RFL sensor for the selective and sensitive detection of 2,4-dichlorophenoxyacetic acid (2,4-D) using the fluorescein nitrobenzoxadiazole (NBD) as a fluorescent source by sol-gel polymerization.

#### 2.1.2. Quantum Dots

Currently, the most used fluorescent nanoparticles in MI-RFL sensors are quantum dots. With higher brightness, chemical stability, biocompatibility, narrow bandwidth, and specific surface area, QDs overcome the problems of organic dyes with low photostability, narrow excitation bands, low signal intensity, and broad emission spectra [33,34]. QDs are usually categorized as semiconductor or carbon-based QDs. Semiconductor QDs consist of elements of groups II–VI, III–V, and IV–VI of the periodic table and are representative of inorganic fluorescent nanomaterials (CdSe, CdS, CdTe, ZnS, etc.). Carbon-based QDs are classified into graphene quantum dots (GQDs), carbon quantum dots (CDs), and carbon nanodots (CNDs) [35]. For example, You et al. [36] constructed an MI-RFL sensor using blue and red CdSe/ZnS QDs grafted on a silica layer as a fluorescence source. Zhu et al. [37] prepared a novel MI-RFL from Mn-ZnS QDs and GQDs@SiO_2_ as a fluorescence source for sensitive and selective visual detection of sinapic acid. Liu et al. [38] constructed MI-RFL using CDs, which was applied to the highly selective and sensitive detection of tetracycline (TC). Zhang et al. [39] encapsulated N, S, B doped CNDs, and red CNDs into MIPs to form MI-RFL for the detection of furazolidone (FZD).

#### 2.1.3. Upconversion Nanoparticles

UCNPs are a special type of nanomaterials mainly composed of oxides or fluorides doped with rare earth elements. Compared with quantum dots, rare earths have the advantages of low cost, low toxicity, low photodamage, high photostability, and strong and sharp fluorescence emission [40]. Currently, UCNPs have been applied in biological and pharmaceutical fields. For example, Shen et al. [41] successfully developed a novel double-MI-RFL sensor based on multilayered core-shell-structured Eu,Tb-doped Y_2_O_3_@SiO_2_ for detecting carbofuran (CF).

#### 2.1.4. Metal Nanoclusters

MNCs are defined as isolated particles less than 2 nm in size containing several to hundreds of metal atoms, which act as a bridge between metal atoms and metal nanoparticles [42]. MNCs, such as AuNCs, AgNCs, and CuNCs, are widely used in various applications due to their excellent properties, such as high fluorescence, high resistance to photobleaching, biocompatibility (compared to organic dyes and toxic QDs), low toxicity, and unique spectrofluorimetric properties [43]. Pirot et al. [44] successfully developed an MI-RFL-based sensor using blue copper nanoclusters (b-CuNCs) and yellow carbon dots (y-CDs) as fluorescent signal sources, with ascorbic acid (AA) cavities printed on ZIF-8.

### 2.2. Working Mechanism of MI-RFL Sensor

MI-RFL sensors work by creating cavities in MIPs polymers that match the shape and function of the template molecules and use these cavities to alter the fluorophore microenvironment by specific recognition of the target and then detect the concentration of the target by the change of the ratiometric fluorescence signal. Regarding ratiometric fluorescence sensors, the target response mechanisms mainly include fluorescence resonance energy transfer (FRET), inner filter effect (IFE), photoinduced electron transfer (PET), and others [45].

#### 2.2.1. FRET

FRET, first proposed by Förster [46], refers to a non-radiative energy transfer process in which the excited-state donor transfers energy to the acceptor when the fluorescence spectrum of the donor overlaps with the excitation spectrum of the acceptor and the spatial distance between the two is sufficiently close [47]. FRET has the advantages of small fluorescence detection error, weak background signal [48], and strong anti-jamming ability, which is conducive to the improvement of ratiometric fluorescence sensors in terms of measurement accuracy and stability, enhancement of sensitivity and resolution, and applicability to complex environments, as well as real-time monitoring and visualization, among other significant advantages, environment, and real-time monitoring and visualization. Fu et al. [49] prepared a novel dual-emission MI-RFL sensor that achieves efficient and visual detection of phycoerythrin (PE) through the FRET mechanism. It provides new tools and strategies for biomolecule detection and promotes the development of high-sensitivity and high-selectivity detection technology.

#### 2.2.2. IFE

As a non-radiative energy conversion process, IFE is usually generated when the absorption spectrum of a light-absorbing substance (the fluorophore itself or other coexisting substances) overlaps with the fluorescence excitation or emission spectrum and the excitation or emission light of the fluorophore is partially absorbed, and sensors based on the IFE can convert absorption signals into fluorescence signals, thus improving the detection sensitivity and selectivity [50,51]. The fluorescence lifetime of the fluorescent groups decreases significantly during the FRET process, whereas the fluorescence lifetime is almost constant during the IFE process [52]. Li et al. [53] successfully synthesized a novel dual-emission MI-RFL sensor (B,N-CDs/ZIF-8@MIP) based on the IFE mechanism to achieve specific and rapid visual detection of doxycycline (DOX). It provides a new idea for efficient, highly selective, sensitive, and intuitive visual detection of coloured substances in complex matrix environments.

#### 2.2.3. PET

A typical PET system consists of a fluorophore and a receptor bound by a spacer group, forming a “fluorophore-spacer group-receptor” model. When the receptor is not bound to the analyte, the fluorophore-spacer group-receptor model loses fluorescence due to the PET process from the receptor to the fluorophore [28,54]. However, when the receptor partially binds to the analyte, the PET action is inhibited or even completely blocked, at which point the fluorophore can emit fluorescence normally upon excitation. Hao et al. [55] constructed a novel MI-RFL sensor based on PET mechanism by sol-gel method for rapid recognition and detection of difenoconazole, which greatly improved the visual detection performance of ratiometric fluorescent sensors and achieved visual detection from fluorescence quenching to fluorescence conversion.

#### 2.2.4. Other Mechanisms

Other mechanisms, such as intramolecular charge transfer (ICT) and aggregation-induced emission (AIE), are less common than the above three but also show unique applications in specific fields. ICT refers to the intramolecular charge transfer of a donor-acceptor after a molecule has been excited by light and is caused by electron conjugation of the electron-donor group with the electron-acceptor group [54]. In contrast to the PET mechanism, the ICT process causes a significant fluorescence band shift, whereas PET quenching does not result in a spectral shift [45]. Miao et al. [56] prepared a novel ratiometric fluorescent sensor for quantitative detection of biogenic amines based on the ICT mechanism. AIE refers to the phenomenon that a class of molecules that do not emit light or emit light weakly in solution emit significantly more light in the aggregated state or in solid films. AIE characteristics have the advantages of low background, good photostability, and high quantum yield [57]. Huang et al. [58] prepared a dual-emission ratiometric fluorescent sensor based on the AIE mechanism for the detection of antibiotics in the environment. It provides a reliable method for quantitatively identifying and distinguishing antibiotics with different or similar structures.

## 3. New Techniques and Strategies for the Preparation of MIPs

The main methods for the preparation of MI-RFL sensors used for biological and pharmaceutical assays in complex samples are free radical polymerization and sol-gel polymerization. In addition to polymerization methods, several novel imprinting techniques and strategies have been developed to improve the performance of MIPs. Novel molecular imprinting strategies include surface imprinting, nanoimprinting, dummy imprinting, multi-template imprinting, and stimulus-responsive imprinting strategies.

### 3.1. Surface Imprinting Technology

Surface imprinting technology involves the preparation of thin layers of polymeric material by controlling the template at or near the surface of the material to create more effective recognition sites [21]. It can overcome the disadvantages of low binding capacity and difficult elution of conventional MIPs. Core-shell structures are the main type of surface-imprinted MIPs and are widely used due to their increased specific surface area and better binding ability. Li et al. [59] constructed a nanoscale core-shell structure, MI-RFL sensor, by surface imprinting using the sol-gel method. This sensor combines the high sensitivity of fluorescence detection with the high selectivity of MIPs for the specific identification and accurate quantification of trace folic acid (FA) in complex matrices.

### 3.2. Nanoimprinting Technology

Nanoimprinting technology prepares nanoscale MIPs. Nanomaterials have a large specific sur face area and specific volume, exposing more binding sites to attract the target analytes and improving the binding capacity of MIPs [60]. Li et al. [61] developed a novel MI-RFL sensor based on nanoimprinting technology for FRET-based thermoregulated sensing and detection of cyanobacteria protein targets. This study provides a simple, fast, and intelligent method for the identification and analysis of trace proteins in complex aqueous matrices and promotes the development of protein imprinting and nanoimprinting-related research.

### 3.3. Dummy Imprinting Strategy

The dummy template imprinting strategy, also known as pseudo-template imprinting strategy, uses compounds that are similar to the target compound in shape, size, structure, and function as templates [22]. It is particularly suitable for target compounds with low abundance (lack of access), high cost, easy degradation, or unstable under polymerization conditions [62]. This strategy can effectively avoid the risk of template leakage and inaccurate detection results. MI-RFL sensors prepared using the dummy template imprinting strategy have been widely used for analytical detection of pharmaceuticals and organisms. Qi et al. [63] used monensin as the fragmentary dummy template molecule and prepared a MI-RFL sensor by precipitation polymerization to detect ciguatoxin P-CTX-3C with high sensitivity and selectivity. Li et al. [64] constructed a highly sensitive MI-RFL sensor for the detection of microcystins (MCs) using metformin as a dummy template. The sensor has good optical properties, water solubility, low toxicity, low cost, and environmental friendliness.

### 3.4. Multi-Template Imprinting Strategy

Multi-template imprinting technology, which uses multiple targets/species as templates, allows for the creation of multiple types of recognition sites in a single polymer material, enabling the efficient removal and enrichment of multiple analytes in a single process, overcoming the potential limitations of single-template MIPs when dealing with multiple impurities [65]. Luo et al. [66] constructed a novel dual-emission MI-RFL sensor based on sol-gel method using a versatile monomer imprinting strategy. The sensor has good sensitivity, selectivity, and anti-interference, which provides a reference for the detection of harmful alkaloids.

### 3.5. Stimulus-Response Imprinting Strategy

Stimuli-responsive molecular imprinting strategy is an approach to prepare MIPs with specific responsive properties. This strategy prepares stimuli-responsive imprinted polymers (SR-MIPs) by introducing stimuli-responsive monomers or elements, such as pH, light, heat, and magnetism, during the preparation process with the help of techniques such as reactive polymerization, click chemistry, and others [67]. Tang et al. [68] designed an acid-sensitive, tri-emitting MI-RFL-based sensor to achieve dual-mode visual detection of ibuprofen (IP), chloramphenicol (CAP), and florfenicol (FF). A new strategy was provided for the detection of multi-target substances in real environments.

## 4. Types of Fluorescence Emission from the MI-RFL Sensor

Currently, MI-RFL sensors include two types: dual-emission and triple-emission. Of these, dual-emission systems are more common due to their relatively simple configuration and wide applicability. The dual-emission system defines the response signal and reference signal by integrating two luminescent substances with different response characteristics so as to achieve dual fluorescence signal output [69]. The output response of the sensor is linearly related to the number of molecules to be measured in a certain range. According to the different nature of the reference signal, MI-RFL sensors can be subdivided into two categories: one is to introduce a second signal that is insensitive to the target as a reference. 

**Figure 3:**
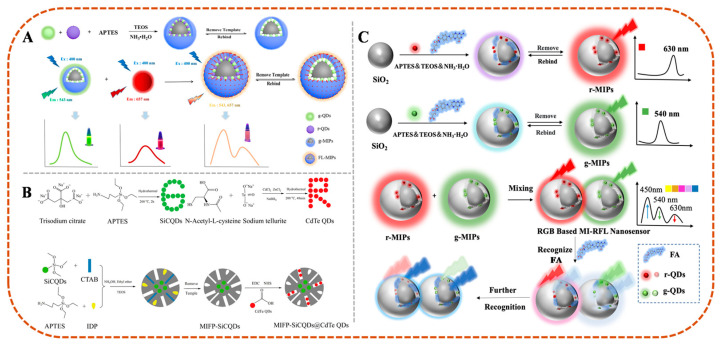
(**A**) The preparation process of green-mission MIPs (g-MIPs) and the construction of dual-emission sensors (FL-MIPs) by PIMix method [16]. (**B**) The synthesis process of SiCDs, CdTe QDs, and MIFP-SiCDs@CdTe QDs by PIMod method [73]. (**C**) Procedure for the preparation of r-MIPs and g-MIPs and the construction of MI-RFL sensors by PIMix method [76].

In the fabrication of MI-RFL sensors, two key technological paths exist, namely PIMix and PIMod. PIMix mainly refers to the process of mixing the imprinted polymer with fluorophores or other functional materials in a certain proportion. The key to this process is to ensure that the components can be evenly distributed and the overall performance of the sensor is stable [15]. PIMod, on the other hand, is a further chemical or physical treatment of an already formed impression polymer to improve its properties or to endow it with new functions. This process reduces background interference, improves recognition selectivity and sensitivity, and enables intra-imprint signal transduction [24,70]. For example, Wang et al. [16] constructed a novel dual-emission MI-RFL sensor to achieve naked-eye detection of bovine serum albumin (BSA) by mixing green-emitting fluorescent MIPs (g-MIPs) and red-emitting CdS/CdTe QDs (r-QDs) in an appropriate ratio using the PIMix strategy. The preparation of MIPs by the PIMix method is shown in Figure 3A. With the increase of BSA concentration, the green fluorescence intensity gradually decreased, while the red fluorescence intensity remained constant. The other is to apply two signal changes in the target response, thus realizing dual-emission ratiometric fluorescence detection, which is a simpler ratiometric strategy with lower background noise and higher signal-to-background ratio [71]. Li et al. [72] constructed a novel MI-RFL sensor for the detection of p-nitroaniline (p-NA) by mixing CDs@MIP and FITC@SiO_2_ in appropriate ratios using the PIMix strategy. Using CDs@MIP as the response signal and FITC@SiO_2_ as the reference enhancement signal, p-NA quenches the fluorescence intensity of CDs@MIP and enhances the fluorescence intensity of FITC@SiO_2_. Wei et al. [73] successfully constructed a dual-emission MI-RFL sensor by anchoring CdTe QDs to multistage mesoporous MIPs as analytical signals via PIMod to improve the detection sensitivity of the conventional doping method for the detection of imidacloprid (IDP) pesticide. The process of preparing MI-RFL sensors via doping and PIMod methods is shown in Figure 3B. 

Although the visualization ability of dual emission is much higher than that of single emission MI-RFL sensor. However, the colour evolution window established by two fluorescence emission peaks is still not wide enough [74,75]. The triple-emission MI-RFL sensor achieves a higher level of sensitivity and accuracy due to its multi-channel signal output, which significantly optimizes the visualization and overall analysis performance. For example, Wen et al. [76] successfully constructed a triple-emission MI-RFL sensor for the visualization and detection of FA in food samples by PIMix r-MIPs and g-MIPs, and the preparation process is shown in Figure 3C. The sensor is promising, and when combined with MIT and skillfully applied with PIMix strategies, the selectivity and sensitivity of the sensor in complex environments can be significantly enhanced, thus further expanding its application in pharmaceutical analysis, biomarkers detection, and environmental monitoring.

## 5. Application of MI-RFL Sensors in Analysis of Pharmaceuticals and Biomarkers

The MI-RFL sensors simulate the specific binding of antigen-antibody in MIT and combine the advantages of anti-interference and fast reaction of ratio fluorescence detection and thereby realize highly specific recognition and accurate quantification of target molecules, for instance, high selectivity and sensitivity in pharmaceuticals and biomarkers analysis [77,78]. It can accurately detect drug residues and biomarkers, so it is widely used in pharmaceutical safety monitoring and life science research. Table 1 summarizes some typical applications of MI-RFL sensors in pharmaceuticals and biomarkers analysis, further highlighting their wide applicability and great potential.

### 5.1. Pharmaceutical Analysis

Pharmaceutical analysis is a key part of ensuring the quality and safety of pharmaceuticals, and MI-RFL sensors can achieve high sensitivity and selective detection of impurities [13,19], residues, and illegal additives in pharmaceuticals. It has been widely used in the detection of trace pesticide residues, veterinary drug residues, and human drug residues in pharmaceuticals. With its high sensitivity, high selectivity, and fast response, MI-RFL sensors realize precise application in pharmaceutical analysis, effectively guarantee food safety, promote precise monitoring and management of environmental pollutants, and maintain human health. 

#### 5.1.1. Detection of Veterinary Drug Residues

Veterinary drug residues refer to the accumulation or residue of the original drug or its metabolites in the body of livestock and poultry or their products (e.g., eggs, milk, and meat) after the use of veterinary drugs. For example, antibiotics such as penicillin G (PNG) and TC residues in food are easy to cause allergic reactions in humans and may even lead to death. Currently, MI-RFL sensors have been widely used in the detection of veterinary drug residues, with most studies focusing on the detection of antibiotics [38,39,53,68,79,80,81,82,83,84,85,86,87,88,89,90,91,92] and hormone drugs [93].

1. Antibiotics

**Figure 4:**
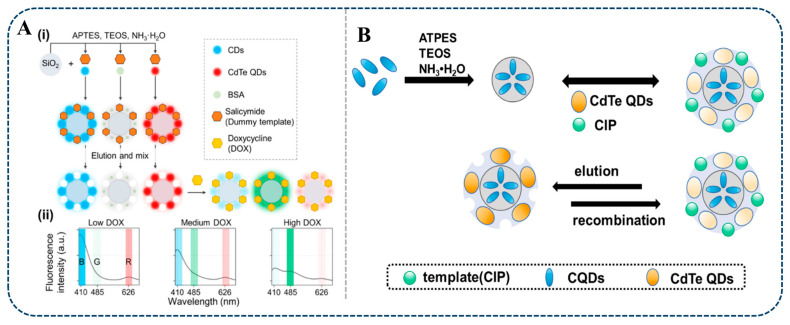
Scheme illustration of the MI-RFL sensors for the detection of veterinary drug residues. (**A**) (i) Preparation strategy of a triple-emission MI-RFL sensor and (ii) detection of DOX [80]. (**B**) Preparation of CdTe/CQDs@SiO_2_@MIPs sensors for detecting CIP [87].

Antibiotic drugs are widely used in animal husbandry, but overuse or misuse may lead to veterinary drug residues, posing a potential threat to human health. Studies have shown that MI-RFL is effective in detecting veterinary drug residues. Miao et al. [80] proposed a novel triple-emission MI-RFL sensor by PIMix strategy using MIPs with different emission fluorescence and salicylamide as dummy templates. Bovine serum albumin (BSA) was selected as a co-functional monomer and as a sensitizer for the AIE effect of DOX. Figure 4A schematically shows the preparation and construction process of this sensor. Blue-emitting CDs and red-emitting CdTe QDs were introduced into MIPs as response signals, respectively. Upon recognition of DOX within 2 min, the blue and red fluorescence of the triple-emitting MIPs sensor was quenched, while the green fluorescence of DOX was enhanced, resulting in the observation of multiple colour variations of blue-violet-red-pink-orange-yellow-green, with the limit of detection (LOD) of 0.061 μM. The sensor, with its highly selective recognition capability, was successfully applied to the detection of DOX in complex real samples, providing great application potential for rapid visual detection of antibiotics in complex matrices. Wang et al. [87] successfully constructed an MI-RFL sensor (CdTe/CDs@SiO_2_@MIPs) for the efficient and rapid detection of ciprofloxacin (CIP) in seawater samples by sol-gel method. The preparation process is shown in Figure 4B. The blue CDs were used as the reference signals, and the orange-yellow CdTe QDs were used as the response signals. The sensor showed good linearity up to 20 μmol/L with a LOD of 0.09 μmol/L and recoveries of 96.2–103.1% in seawater. After pretreatment with solid phase microextraction, the sensor was successfully applied to the determination of CIP in seawater. Under the 365 nm ultraviolet light, the sensor showed a green-blue colour change window visible to the naked eye. The successful preparation of this sensor provides a simple, versatile, and visible alternative method for the detection of CIP in seawater. Therefore, the MI-RFL technique is expected to be used for rapid in situ visual detection of various target analytes and to promote the development of MI-RFL technique.

2. Hormones

Hormonal drugs are mainly used to improve the reproductive and productive performance of animals, such as sex hormones like estradiol. Residues of such pharmaceuticals in animal food can interfere with hormone metabolism and physiological functions of consumers, which is potentially hazardous to human health. Wang et al. [93] used 17β-estradiol (E2) as the template and PET as the response mechanism, mixed blue fluorescence 7-hydroxycoumarin (b-MIPs) and yellow fluorescence CdTe QDs(y-MIPs) based on PIMix strategy to construct a new dual-emission MI-RFL sensor for the detection of E2. The results showed that the sensor had good linearity in the concentration range of 0.011–50 μg/L, with the LOD of 3.3 ng/L, the recoveries of 92.4–110.6%, and the relative standard deviation (RSD) less than 2.5%. When E2 was detected by the sensor, yellow fluorescence quenched, blue fluorescence increased, providing a green-blue visual monitoring window visible to the naked eye. The specificity and sensitivity of the sensor, as well as the self-correction and visualization of the dual signals, open more possibilities for future development of multimodal visual sensing systems. How to effectively determine the mixing ratio of dual-emission ratio fluorescence sensors is also an issue that needs further exploration.

#### 5.1.2. Detection of Pesticide Residues 

Long-term and large-scale use of pesticides can result in pesticide residues in agricultural products, which include trace pesticide precursors, toxic metabolites, degradation products and impurities, and so forth, and human ingestion may trigger chronic poisoning, posing a potential threat to health. In addition, this overuse seriously disrupts the balance and stability of the ecosystem, exacerbates food safety problems, and endangers human health [118]. Currently, MI-RFL sensors have been widely used for the detection of pesticide residues, with most studies focusing on the detection of insecticides [36,41,95,102,103,119], herbicides [32,78,94,96,98,99,101,104]. and bactericides [92].

1. Insecticides

**Figure 5:**
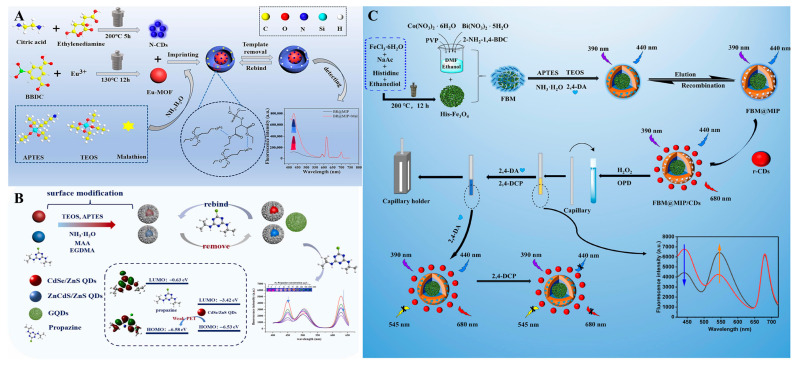
Scheme illustration of the MI-RFL sensor for the detection of pesticide residues. (**A**) Preparation and construction process of MI-RFL sensor (N-CDs@Eu-MOF@MIP, BR@MIP) for the detection of MAL [120]. (**B**) Construction process of a novel MI-RFL sensor for the detection of propazine [96]. (**C**) Procedure for the construction of a triple-emission MI-RFL sensor for the detection of 2,4-D and 2,4-dichlorophenol [101].

Insecticidal pesticides are chemical substances used to prevent, destroy, or control pests. In the application of pesticide residue detection using the MI-RFL sensor, the insecticides detected can be subdivided into several types, such as pyrethroids, carbamates, organophosphates, and neonicotinoids. Carbaryl is widely used as a carbamate insecticide, and its improper use can lead to pesticide residues, which pose a significant threat to human health. Wang et al. [119] constructed an MI-RFL sensor based on fluorescence-enhanced APTES@CdTeS QDs based on the response mechanism of PET, which was used for the determination of 1-naphthol (1-NP), the synthetic raw material and metabolite of the pesticide carbaryl. The sensor showed a good linear relationship between the fluorescence intensity ratio (F_460_/F_597_) and 1-NP concentration over a wide concentration range (6.0–140.0 μM, LOD = 0.45 μM, RSD < 4.41%). The fluorescence of the sensor changed from orange-red to blue-violet and was clearly visible. The method was applied to detect 1-NP in traditional Chinese medicine (TCM) products with good recoveries, demonstrating its potential for the detection of 1-NP and carbaryl. In addition, the simple modification of QDs materials by APTES for fluorescence enhancement can expand the responsiveness of the target object, which is very meaningful for fluorescence analysis, and more detection systems can be developed based on this method. The organophosphorus pesticide malathion (MAL) is a broad-spectrum insecticide. However, MAL has the toxicity of inhibiting neurotransmitters, and its overuse can be hazardous to human health. Yang et al. [120] constructed a novel MI-RFL sensor (N-CDs@Eu-MOF@MIP, BR@MIP) for the selective identification and sensitive detection of MAL. N-doped carbon dots (N-CDs) act as a fluorescence source to generate fluorescence signals, and the fluorescence colour of the sensing system varies from red to blue, which is helpful for visual analysis. The specific synthesis process of the N-CDs@Eu-MOF@MIP sensor is shown in Figure 5A. MAL showed good linearity over 1–10 μM with a LOD of 0.05 μM. In addition, the recovery of the sensor in real samples was 93.0–99.3%. In addition, the smartphone-based sensor was used to detect MAL in simulated real samples, and a good practical application of the fluorescence sensor was achieved. Therefore, this MI-RFL sensor construct provides a good strategy for MAL detection.

2. Herbicides

Herbicides are agents that destroy or inhibit plant growth by causing complete or selective weed death. For example, promazine is a widely used triazine herbicide., and its residue detection is important for agricultural cultivation and prevention of potential risks to human health. Promazine is a widely used triazine herbicide. Given its stability, resistance to degradation, and long half-life, it can accumulate in crops and can pose risks to, inter alia, human health and ecosystems. Liu et al. [96] prepared a novel MI-RFL sensor based on the PET mechanism for selective detection of propazine. Firstly, r-MIP-QDs and b-MIP-QDs were synthesized by inverse microemulsion method using CdSe/ZnS QDs (red light) and ZnCdS/ZnS QDs (blue light). b-MIP-QDs, r-MIP QDs, and GQDs (green light) were mixed in an optimal ratio, and the construction and detection of the sensor are shown in Figure 5B. At present, machine learning algorithms have been used to analyze complex fluorescence data, to realize the rapid identification and quantitative analysis of target molecules. This experiment integrates the support vector regression algorithm and the three-colour perception platform to facilitate model training on existing data and realize pattern recognition and prediction of unknown colour values. The recoveries of this sensor were 104.0–114.6% and 92.0–96.4%, respectively, and the LODs were 5.0 μg/kg and 1.0 μg/L. There should be a significant colour change (from rose to light orange, from dark orange to olive, and ultimately to dark green) for the different concentrations of prochloraz, showing excellent sensitivity. The sensor is of great practical importance as a rapid detection method. Chen et al. [101] constructed a three-emission MI-RFL sensor for the simultaneous detection of 2,4-D and 2,4-dichlorophenol by sol-gel method. The FBM@MIP was prepared by grafting a nano-enzymatic fluorescent organic framework (Bi, Co-MOF) on the histidine-modified Fe_3_O_4_ surface to form a nano-enzymatic complex (FBM) with dual enzyme activity and imprinting the FBM@MIP with 2,4-D. The specific preparation flow of the sensor is demonstrated in Figure 5C. The linear concentration ranges were 1.0 × 10^−12^–1.2 × 10^−9^ M and 1.0 × 10^−12^–4.8 × 10^−9^ M, with LODs of 0.75 and 0.68 pM, respectively. And combine with a smartphone to provide a new method for intelligent sensing of multiple targets at the same time, which provides an ultrasensitive and novel strategy for the simultaneous detection of multiple pollutants in a complex environment.

3. Bactericides

In agriculture, bactericides are a class of pesticides used to control plant diseases caused by various pathogenic microorganisms. Ma et al. [92] constructed a new MI-RFL sensor and used greed fluorescence (NBD-APTES) as the control fluorescence source to realize the rapid visual detection of difenoconazole by using the fluorescence changes of cysteine-modified CDs (CDs-Cys). Under the optimized conditions, the linear range of the fluorescence sensor was 0.3–60 μmol/L, the LOD was 75 nmol/L, and the sample recovery was 102.1–111.2%. The sensor is highly specific and has been successfully applied to the detection of real samples. Therefore, this sensor can be used as an analytical tool for difenoconazole and provide a basis for future research on the rapid detection of different pesticide residues in the field. Compared with the current single fluorescence sensor, the combination of ratio fluorescence greatly improved the visual detection performance of the sensor and realized the visual detection from fluorescence quenching to fluorescence conversion. 

#### 5.1.3. Pharmaceuticals for Human Use 

Analytical research on human pharmaceuticals plays an important role in safeguarding public health and safety. With the advancement of modern medical technology, accurate analysis of pharmaceutical components has become particularly important to optimize drug formulations, enhance therapeutic efficacy, and accelerate the development of new pharmaceuticals. However, human pharmaceuticals also have drug residues, which may cause serious impacts on human health and the environment if not handled properly or used in excess [121]. In this context, MI-RFL sensors, as an advanced detection technology, have been widely used in the analysis and research of human pharmaceuticals, especially in the accurate analysis of natural organic compounds, complex systems of TCM [66,106,111], and chemically synthesized pharmaceuticals [44,59,68,76,108,109,110,112,113,121], showing great potential.

1. Natural Organic Compounds and TCM

**Figure 6:**
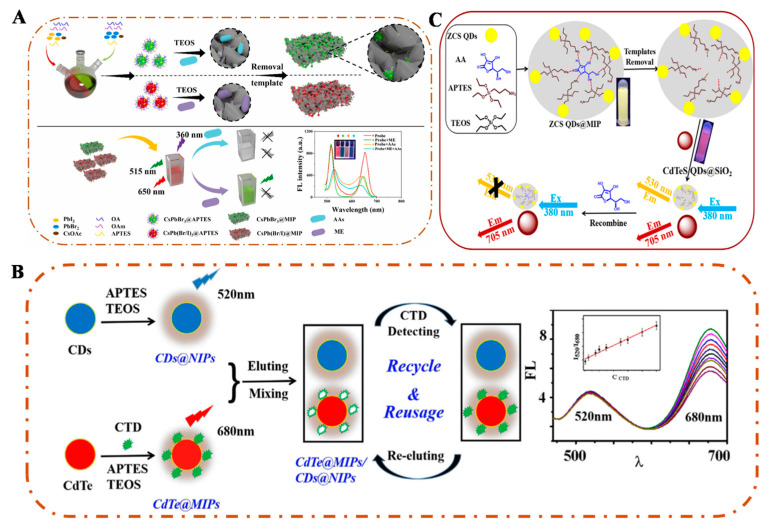
Scheme illustration of the MI-RFL sensor for the detection of pharmaceuticals for human use. (**A**) Preparation process of a dual-emission MI-RFL sensor for the detection of MEs and AAs [106]. (**B**) Procedure of the main method for CTD detection in blood based on MI-RFL sensor [111]. (**C**) Schematic illustrations of ZnCdS QDs@MIP/CdTeS QDs@SiO_2_ sensor for the recognition of AA [112].

Natural organic compounds are organic substances widely found in nature and synthesized by living organisms, while TCM are medicines based on these natural organic compounds and applied under the guidance of traditional Chinese medical techniques, including botanicals and animal medicines. Nowadays, MI-RFL sensors have been widely used in the field of natural organic compounds and TCM detection. Asarum belongs to the genus Asarum of the Aristolochiaceae family and contains methyl eugenol (ME) and aristolochic acid A (AAs), which have a wide range of pharmacological activities but have a certain degree of hepatotoxicity and so forth. In order to detect the harmful components in TCM, Du et al. [106] employed a multi-template imprinting strategy with AAI and ME as template molecules and two PQDs, CsPbBr^3^ and CsPb (Br/I)^3^, as the fluorescence sources and constructed a novel dual-emission MI-RFL sensor based on the sol-gel method and PET response mechanism for the selective and sensitive detection of ME and AAs in *Aristolochia* spp. The construction and detection process of the sensor is shown in Figure 6A. The sensor has dual emission peaks at 515 nm and 650 nm, and the ME can only quench the emission peak at 650 nm, while the AAs can quench both emission peaks. Therefore, the presence of ME, AAs, or both can be qualitatively identified based on the different colours of the sensor. In addition, the fluorescence intensity ratios at 650 nm and 515 nm showed a good linear relationship with the concentrations of ME and AAs, with LODs of 8.3 nM for ME and 9.6 nM for AAs. The good selectivity of the sensor for the determination of ME and AAs and the obvious colour changes in visual detection provide valuable insights for the rapid detection of active ingredients in TCM. Canthaxanthin (CTD) is an active ingredient of TCM with unique therapeutic effects, but it may lead to poisoning or death due to improper use. Therefore, an analytical method is needed to monitor the concentration of CTD in biological samples. Ling et al. [111] successfully constructed a dual-emission MI-RFL sensor (CdTe@MIPs/CDs@NIPs) for the selective detection of CTD by using a sol-gel method, in which CdTe@MIPs and CDs@NIPs were synthesized separately, and the construction flow is shown in Figure 6B. The CDs@NIPs were directly used as the reference signals, while the prepared CdTe@MIPs were used as the response signals. After the addition of CTD, the fluorescence intensity of CdTe@MIPs decreased, while that of CDs@NIPs remained largely unchanged, and the LOD was as low as 0.15 nM. The sensor was successfully applied to the detection of CTD in human blood samples with recovery rates between 96.12–107.40% and RSD between 2.87–3.96%. The recyclable ratio fluorescence sensor designed in this study has the characteristics of low cost, simple operation, and high sensitivity, which has broad application prospects in the detection of CTD and is expected to lay the foundation for the application of poison sensing in forensic toxicology analysis.

2. Synthetic Pharmaceuticals

Chemically synthesized pharmaceuticals are compounds with specific pharmacological activity synthesized through a series of chemical reactions using organic or inorganic compounds as raw materials. Although chemically synthesized pharmaceuticals are fast and effective in the treatment of diseases, they are often accompanied by different degrees of side effects, and the MI-RFL sensor can achieve sensitive and accurate detection of chemically synthesized pharmaceuticals and their metabolites. Methotrexate (MTX) is a chemotherapeutic pharmaceutical that is widely used in the treatment of various cancers, but the use of MTX is often accompanied by side effects such as hepatotoxicity and cardiotoxicity, and the therapeutic range of MTX is very narrow, with high doses being life-threatening [122]. Therefore, therapeutic pharmaceutical monitoring is an effective tool to ensure the efficacy and safety of MTX therapy. Alanazi et al. [108] successfully synthesized a novel highly selective and reliable dual-emission MI-RFL sensor based on blue emissive fluorescent CDs (AS-CDs) and IFE as response mechanism using APTES as a functional monomer and TEOS as a cross-linking agent for accurate, sensitive, and simple quantitative detection of MTX. The sensor was based on the measurement of emission ratio (F_355_/F_430_) with a detection range of 5–2000 ng/mL and a LOD of 1.5 ng/mL with satisfactory recoveries. In conclusion, this study provides a method with good selectivity and sensitivity for the detection of MTX in real samples. AA is involved in many important biological processes in vivo, so its detection is of great value in the diagnosis and treatment of diseases. Yang et al. [112] constructed a triple-emission novel MI-RFL sensor based on a sol-gel method with APTES as a functional monomer and TEOS as a cross-linking agent for the specific recognition and sensitive detection of AA. The construction process of this sensor is shown in Figure 6C. In this sensor, CdTeS QDs@SiO_2_ was used as the reference signal, and ZnCdS QDs@MIP was used as the response signal, which improved the accuracy of the measurement results. The fluorescence intensity ratios (F_530_/F_705_) of ZnCdS QDs@MIP and CdTeS QDs@SiO_2_ showed a good correlation with the concentration of AA in the range of 1–500 μM, and the LOD was 0.78 μM. The reliability of the proposed sensor for the analysis of real samples was investigated by detecting AA in vitamin C tablets, and satisfactory results were obtained. This study provides a new strategy for the establishment of MI-RFL sensors based on QDs, which has a promising application in the field of fluorescence sensing.

### 5.2. Biomarkers Analysis

Bioanalysis mainly focuses on the analysis of biomarkers, including proteins, nucleic acids, and metabolites. These biomarkers are widely involved in key physiological processes, such as cell-to-cell signaling, metabolic regulation, and immune response. Therefore, the precise isolation of these substances from biological samples is extremely important for understanding their physiological functions and pathological mechanisms and developing related disease prevention, diagnosis, and treatment strategies [69]. Currently, MI-RFL sensors have been widely used for the precise analysis and sensitive detection of protein, nucleotide, and amino acid biomarkers [16,17,26,49,114,115,116,117]. 

#### 5.2.1. Proteins

**Figure 7:**
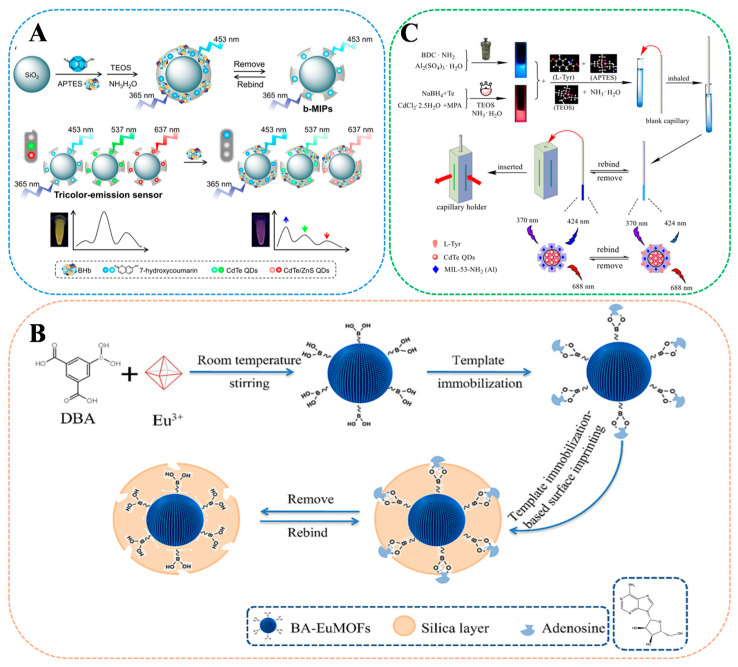
Scheme illustration of the MI-RFL sensor for the detection of biomarkers. (**A**) Schematic diagram of the construction of a triple-emission MI-RFL sensor for checking BHb [117]. (**B**) Schematic of the synthesis process and detection of the MI-RFL sensor (MOF@CdTe@SiO_2_@MIP) capillary for detection of AD [115]. (**C**) Synthetic procedure of MI-RFL sensor for L-Tyr detection [17].

Protein biomarkers, as indispensable biomolecules in living organisms, have complex biological activities and are widely involved in key life processes, such as intercellular signaling, enzyme catalysis, and immune reactions. He et al. [116] prepared a novel dual-emission MI-RFL sensor (SiO_2_@CDs&NBD@MIP) for the selective and sensitive detection of C-type natriuretic peptide (CNP) in biological samples by sol-gel polymerization using NBD as a sensitive signal source and CDs as a reference signal. Based on the PET strategy, CNP could quantitatively enhance the fluorescence intensity of NBD, while the fluorescence intensity of CDs remained unchanged. The sensor combines the advantages of MIPs and ratiometric fluorescent materials and exhibits specific recognition and sensitive detection of CNP with good stability. The linear range of CNP determination was 580 pg/mL, and the LOD was 2.87 pg/mL. Finally, the sensor was successfully applied to the determination of CNP in human serum samples, with the recoveries as high as 97.3–104%, and the RSD was lower than 4.7%. The results indicate that this method is promising for the detection of trace peptides in complex matrices and has great potential in healthcare and public safety. Yang et al. [117] embedded three MIPs in blue-emitting 7-hydroxycoumarin, green-emitting CdTe QDs, and red-emitting CdTe/ZnS QDs, respectively, by sol-gel. b-MIP, g-MIP, and r-MIP obtained were imprinted and mixed in an optimal ratio to construct a triple-emission MI-RFL sensor for the detection of bovine hemoglobin (BHb), and the construction and detection process is shown in Figure 7A. This triple-emission sensor recognizes BHb with simultaneous extinction of green and red fluorescence and simultaneous illumination of blue fluorescence, thus generating a significant concentration-dependent green-red-blue window for visual detection of BHb by the naked eye. The fluorescence intensity varied linearly in the range of 0.025–3 μM with a LOD of 7.8 nM, and the bovine recoveries were as high as 99.25–111.7%. The optical stability was good, and the post-imprint construction was convenient. It was demonstrated that the sensitivity, visualization, and working efficiency of the triple-colour emission fluorescence MIPs sensor were significantly improved by integrating the surface imprinting, triple-colour emission fluorescence, and post-imprint hybrid structures.

#### 5.2.2. Nucleotides

Nucleotides play important physiological functions in organisms, among which adenosine (AD) belongs to the nucleotide class of biomarkers, and the development of highly selective and sensitive AD detection methods is important for the early diagnosis of clinical diseases. Cheng et al. [115] designed a simple, fast, and highly sensitive novel dual-emission MI-RFL sensor based on FRET mechanism, capable of dual recognition of adenosine. Figure 7B shows its synthesis route. The strategy used in this study was to combine boronic acid functionalized lanthanide metal-organic frameworks (BA-EuMOFs) with MIPs. Among them, BA-EuMOF not only provides a stable and sensitive ratiometric fluorescence recognition signal for BA-EuMOFs@MIP but also generates fluorescence signal changes by FRET for signal amplification. Based on dual recognition sites, the constructed ratiometric fluorescence sensor showed satisfactory photostability, sensitivity, and selectivity in AD analysis. The BA-EuMOFs@MIP sensor had a wide linear range (1–50 mg/L) and a sensitive LOD (0.26 mg/L) for AD. AD in real samples was detected using this sensor, with recoveries ranging from 96.11–101.79%. This method not only provides a simple, rapid, and sensitive sensing platform for AD detection but also shows great potential for early diagnosis of AD-related diseases.

#### 5.2.3. Amino Acids 

Amino acids are the basic building blocks of proteins, and long-term intake can lead to dependence, affecting natural metabolism and regulation, as well as potentially triggering diabetes or kidney problems. Tang et al. [17] constructed a novel dual-emission MI-RFL sensor based on a luminescent metal-organic framework (MOF@CdTe@SiO_2_ @MIP capillary) through the IFE mechanism and surface imprinting strategy for chiral recognition of L-Tyr. Figure 7C illustrates the synthetic procedure and detection. Firstly, (MIL-53-NH_2_ (Al) and CdTe@SiO_2_ were embedded as a fluorescence source into the imprinted layer grown on the inner surface of amino-modified capillaries by hydrothermal and sol-gel methods. In the presence of L-Tyr, the blue fluorescence (424 nm) as the fluorescence response signal was quenched, while the red fluorescence (688 nm) as the reference signal remained unchanged. The MI-RFL sensor successfully detected L-Tyr in real samples in a linear range of 1.0 × 10^−10^–2.5 × 10^−8^ M with recoveries of 99.07–101.18% and a LOD of 8.0 × 10^−11^ M. The sensor integrates MIT and fluorescence capillary method to achieve sensitive and selective determination of L-Tyr with a small amount of reagent consumption (18 μL), providing a new strategy for the rapid detection of trace samples with simple and environmentally friendly operation.

## 6. Application of MI-RFL Sensors for POCT

Traditional analytical methods usually rely on large-scale precision instruments, which have the disadvantages of cumbersome operation and high cost and so on. Due to the urgent demand for rapid and accurate detection methods in the fields of environmental monitoring, food safety, and modern medical treatment, rapid detection methods have become an important hotspot for scientific research and technology applications [123]. In this context, MI-RFL sensors have been widely used in POCT, which emphasizes rapid analysis at the sample collection site and instant access to test results using portable instruments, and is simple, cost-effective, and portable, which greatly enhances the efficiency and convenience of testing. The MI-RFL sensor combines the high selectivity of molecular imprinting technology for specific target molecules with the high sensitivity and real-time monitoring capability of fluorescence detection technology to achieve rapid and accurate detection of a wide range of analyses, such as biomarkers, drug residues, environmental pollutants, and so on. Currently, MI-RFL sensors have been widely used in the POCT field, mainly including test strips [79,96,124,125], smartphones [52,53,68,79,95,101,102,103], and microfluidic chip [55,78,105] mode in various forms, which meets the detection needs in different scenarios and promotes the rapid development of instant detection technology.

### 6.1. Test Strips

**Figure 8:**
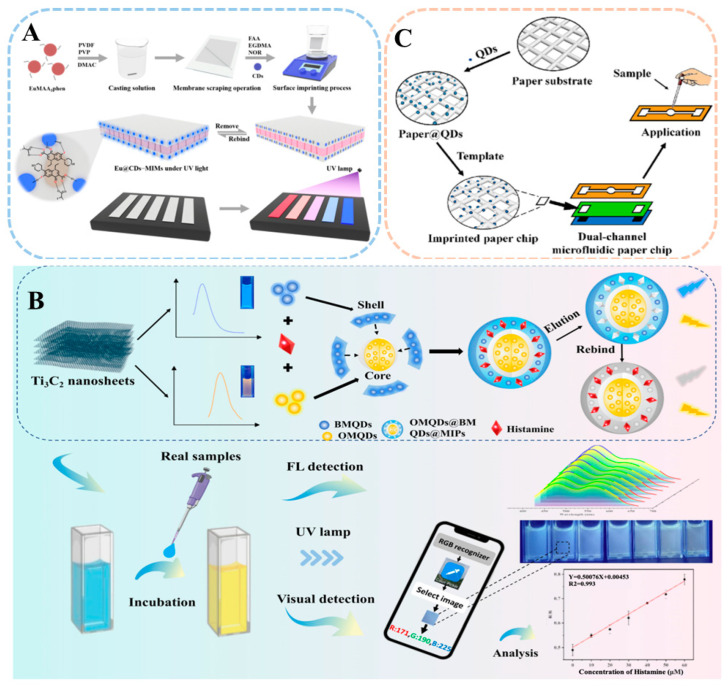
Schematic illustration of the MI-RFL sensor applied to POCT. (**A**) Synthesis of novel fluorescent detection strips (Eu@CDs-MIMs) for the detection of NOR and visual inspection process [79]. (**B**) Preparation and detection process of MXene QDs-based smartphone-driven MI-RFL sensor for the detection of histamine [114]. (**C**) Preparation procedure of dual-channel microfluidic paper chip for detection of difenoconazole [105].

Fluorescent test strips can absorb specific wavelengths of light and emit fluorescence, achieving the detection of target substances by detecting the change of fluorescent signals, with high selectivity, high sensitivity, and high self-correcting, and can be observed with the naked eye under ultraviolet light for colour changes. In recent years, the composition and morphology of nanoparticles embedded in test strips have expanded from AuNCs to a variety of complex nanostructures [126], dramatically improving detection sensitivity, specificity, and stability. These innovations have accelerated the development of POCT technology, while fluorescent test strip assays have also been used in conjunction with MI-RFL sensors for pharmaceuticals and biomarkers detection due to their portability and cost-effectiveness. Hu et al. [79] successfully developed a novel “turn-on” fluorescent test strips (Eu@CDs-MIMs) on PVDF membranes doped with Eu complexes for selective, rapid, and on-site visual detection of norfloxacin (NOR). The synthesis method and the procedure of visual detection is shown in Figure 8A. The response time of the test strips is 1 min. The portable fluorescent test strips have a brighter and more uniform fluorescent colour change than filter paper test strips, which is visible to the naked eye, providing a reliable environmentally safe method for the rapid, visual, and on-site detection of NOR and quinolones.

### 6.2. Smartphones

With the advantages of easy operation, fast processing speed, and high resolution, smartphones have attracted much attention in the field of POCT sensing [120]. It captures fluorescence photos through the built-in camera and analyses them through software, thus providing a low-cost, portable monitoring platform for on-site detection. Currently, a series of smartphone-based fluorescence sensor strategies have been designed and applied for the determination of various targets [58]. In particular, the smartphone-based MI-RFL sensing platform is more conducive to accurate quantitative monitoring due to its intrinsic calibration function. As shown in Figure 8B, Wang et al. [114] prepared a smartphone-driven MI-RFL sensor based on blue-orange MXene QDs by sol-gel method and based on the PET response mechanism for fluorescence and visual detection of histamine. The results showed that the histamine concentration was linearly related to the fluorescence response of the sensor in the range of 1–60 μM, and the fluorescence detection had a LOD of 21.9 nM and a visual LOD of 92.2 nM. The recoveries of this method were in the range of 96.52–105.32%. Considering the on-site quantification requirements in practical applications, the system was connected to a smartphone-based application for image acquisition and red-green-blue (RGB) analysis. Thus, this study greatly expands the application of MXene QDs in fluorescence sensing and provides a visualization strategy for the in situ detection of histamine in food.

### 6.3. Microfluidic Chips

Microfluidic analysis technology is an advanced technology for precise manipulation and processing of fluids on a micro-scale, known for its advantages of miniaturization, high integration, low sample consumption, and fast response [127]. Among them, microfluidic paper chip is an innovative form of microfluidic technology, which uses paper as the substrate material and constructs microfluidic channels on the paper chip to enable the target to move robustly and automatically through capillary action, thus realizing rapid on-site detection. Microfluidic paper chip technology has the advantages of low cost, portability and ease of use, multi-channel detection, and others [128]. Combined with MI-RFL technology, it can provide good visual detection capability and has a wide range of application potential in biomedical detection, environmental monitoring, and other fields. 

Hao et al. [105] used L-Cys-modified CQDs and CdTe QDs as fluorescent signals to prepare a novel dual-channel, dual-signal microfluidic paper chip that enabled portable and rapid detection of difenoconazole by means of an IFE response mechanism. The preparation and detection procedure is shown in Figure 8C. The results show that rapid quantitative detection of the two templates can be achieved within 5 min at the same excitation wavelength (360 nm). In addition, the dual-channel microfluidic paper chip also showed good specificity for the identification of analogs. This work successfully prepared a multi-template rapid quantitative pesticide residue detection sensor, which provides a useful exploration for the development of new general rapid detection technology and theory and is of great significance for the rapid detection of pesticide residues in export products.

## 7. Conclusions and Prospects

As mentioned above, MI-RFL sensors have demonstrated significant advantages and wide application potential in the field of pharmaceuticals and biomarkers analysis. Such sensing methods successfully convert invisible molecular recognition into colour-visible fluorescence signals, which can accurately and quickly detect target molecules in complex environments, providing important support for human health, environmental monitoring, and so forth. Although MI-RFL sensors have achieved remarkable results in the detection of pharmaceuticals and biomarkers, they still face some challenges and promising opportunities, so we make an outlook for their future development.

(1) Sensor construction and optimization: The application of MI-RFL sensors in pharmaceuticals and biomarkers detection depends on their efficient, sensitive, and selective performance, and the optimization of the construction process is critical to achieve these performances. The construction process of MI-RFL sensor mainly includes the selection of fluorescence signal sources and the preparation of MIPs.
  (i) Selection of fluorescence source: Although traditional fluorescent dyes and nanomaterials have been widely used, issues such as stability, spectral overlap, toxicity, and cost still need to be addressed. Cyanine dyes have excellent photostability, wide spectral range, and tunable fluorescence characteristics. Meanwhile, the relatively high quantum yield of cyanine dyes helps to improve the detection sensitivity of the sensor. These advantages make it potential for application in the field of MI-RFL sensors [129,130]. Therefore, the development of fluorescent dyes, such as cyanine dyes, and new fluorescent materials, such as BU-MOFs, and the optimization of their optical properties through surface modification, doping, and recombination become an important direction of future research. At the same time, the integration strategy of multiple fluorescence sources is also expected to achieve effective cooperation between different sources and improve the detection accuracy.
  (ii) Selection of reaction mechanism: In practical applications, the appropriate reaction mechanism should be selected according to the specific detection requirements and the nature of the target molecules. For drugs or biomarkers with specific structures and properties, mechanisms such as FRET, PET, and ICT can be selected to achieve highly selective and responsive detection by designing specific fluorescent probes and receptors. In the pursuit of rapid detection, the IFE mechanism is more advantageous because it does not require a complex energy transfer process and can respond to the target faster. For high sensitivity detection requirements, PET and AIE mechanisms may perform better, which achieve significant fluorescence changes at low concentrations [131]. Furthermore, the combination of multiple reaction mechanisms and sensor design strategies can improve the performance and applicability of the sensors. In the future, with deepening understanding of different reaction mechanisms, reaction mechanisms can be precisely selected and designed to further improve the performance of MI-RFL sensors [132].
  (iii) Preparation of MIPs: The MIPs preparation process was optimized, including the selection of template molecules, polymerization conditions of functional monomers, type and dosage of cross-linking agents, and others, which could significantly improve the performance of the sensor. In addition, the introduction of dummy templates, multi-template strategies, and new imprinting methods, such as electrochemical imprinting and light-controlled imprinting, also provide the possibility to further improve the performance of sensors. The application of theoretical calculations during the preparation of imprinted materials will be an important research direction. For example, Chi et al. [133] used density functional theory (DFT) to calculate the binding energy and molecular electrostatic potential of template molecules and functional monomer complexes and fabricated an MI-RFL sensor with excellent performance for the detection of mycotoxins in food under the guidance of theoretical calculations. This example shows that by simulating the interaction between template molecules and functional monomers, the optimal polymerization conditions can be determined. Then the performance of MI-RFL sensors can be effectively improved. In the future, theoretical computing will also have broad application prospects in the field of pharmaceuticals and biomarkers, providing strong technical support for accurate detection, precise diagnosis, and treatment.
(2) Introduction of new functional materials: The introduction of new functional materials, such as MOFs, covalent organic framework (COFs), and hydrogen-bonded organic framework (HOFs) can bring higher sensitivity and selectivity to MI-RFL sensors. These materials have unique physicochemical properties, such as high specific surface area, tunable pore size, and excellent chemical stability, which can significantly enhance the performance of the sensors. Currently, MOFs are widely used in the construction of MI-RFL sensors, while COFs are relatively less used. This is because COFs have some problems, such as their strong π–π stacking interactions, which lead to quenching effects, and their synthesis is difficult and requires strict reaction conditions, while there are also problems in stability and compatibility in practical application scenarios [134,135]. HOFs are still in their infancy, and their applications are limited. On the one hand, due to the late start of their research, people’s understanding of their physicochemical properties and application potential is not comprehensive. On the other hand, HOFs are ordered porous materials formed by intermolecular hydrogen bonding assembly. Their structural stability is easily affected by surrounding factors, and they face many technical difficulties in achieving high selectivity for specific analytes, which is the main reason why their development lags far behind other porous materials such as MOFs and COFs [136,137]. In the future, with the deepening of research on MOFs, COFs, and HOFs, these materials will play a greater role in the fields of biosensing, environmental monitoring, and drug delivery.
(3) Currently, the research on dual-emission MI-RFL sensors is relatively mature, while the research on triple-emission ratiometric fluorescence sensors is still insufficient but has great potential. PIMix and PIMod, as an ideal construction strategy, effectively enhance the sensor performance by optimizing the material ratios and surface modification. However, the triple-emission technology also faces the problems of complicated preparation process, high cost, and stability issues, such as fluorescence intensity attenuation of multiple emission peaks and shifting of emission peaks, which need to be solved by subsequent research. In the future, it is expected that the triple-emission ratiometric fluorescent sensors will be further developed in the field of pharmaceuticals and biomarkers analysis.
(4) At present, the application and research of MI-RFL sensors in the field of green imprinting technology is in the embryonic stage but has shown great potential and broad prospects to lead the trend of greening chemical sensors in the future. Green imprinting technology not only runs through the entire life cycle of MI-RFL sensors from design, preparation, to use, but also aims to significantly reduce chemical waste, lower energy consumption, and significantly improve the environmental friendliness of the sensors through environmentally friendly and sustainable strategies.
(5) In the field of pharmaceuticals and biomarkers analysis, MI-RFL sensors, although showing advantages such as high selectivity and sensitivity, still face many challenges. The main challenges include improving the sensor’s immunity to interference in complex samples, optimizing the material preparation process to ensure reproducibility, and overcoming the difficulty of template molecule removal. In addition, there is a need to further improve the selectivity and sensitivity of MI-RFL sensors for detecting trace residues in complex matrices, as well as to effectively overcome the interference caused by matrix effects, to enhance their ability to detect target molecules at low concentrations. Therefore, future technological innovation will be the key, including the introduction of new functional materials, optimization of the sensor structure, simplification of the sample pre-treatment process, and realization of multi-component simultaneous detection and so on, which will promote the wide application of MI-RFL sensors in the field of pharmaceutical analysis.
(6) Current MI-RFL sensors have been closely integrated with POCT technology to achieve more convenient and visualized on-site detection. Through the integration with smartphones, test strips, microfluidic paper chips, and other technologies, the sensor can perform rapid and accurate testing directly on-site, which greatly improves testing efficiency and convenience. In the future, with the continuous progress of machine learning and theoretical calculation technologies, MI-RFL sensors are expected to achieve deep integration with these advanced technologies on the existing basis to further improve the detection accuracy and intelligence. This will provide more efficient and accurate solutions for medical and healthcare, environmental monitoring, and other fields.

In summary, MI-RFL sensors show remarkable potential and application prospects in the field of pharmaceuticals and biomarkers detection. In the face of the current challenges, such as material selection, preparation process, sensitivity and stability, and others, which need to be further explored and innovated, the research community is actively seeking innovative breakthroughs through the introduction of new materials, new technologies, and greening and intelligent strategies to promote the development of MI-RFL sensors in a more efficient, environmentally friendly, and sustainable manner. In the future, with the continuous maturation of the technology and the deepening of interdisciplinary cooperation, MI-RFL sensors are expected to realize accurate and efficient detection in more fields and make more excellent contributions to human health, environmental safety, and scientific and technological progress.

## Figures and Tables

**Table 1:** Application of MI-RFL Sensors in the Analysis of Pharmaceuticals and Biomarkers.

Type	Analyte	Fluorescence Sources	Functional Monomer	Crosslinker	Polymerization Method	Working Mechanism	Imprinting Strategy	Linear Range	LOD	Real Sample	Recovery (%)	RSD (%)	Ref.
Pharmaceuticals analysis	NOR	CDs	MAA	EDGMA	–	ICT	Surface imprinting	5–50 nM	1.35 nM	Tap water, river water	93–106.8	2.0–3.6	[79]
	DOX	CdTe QDs	APTES	TEOS	Sol–gel method	IFE	Dummy template imprinting	0.1–50 μM	0.061 μM	Seawater, reservoir water, urine	91.6–107.5	2.9–4.6	[80]
		CDs	APTES	TEOS	Sol–gel method	AIE, IFE	–	0.05–20 mg/L	14.21 ng/mL	Lake water, milk	90.16–97.18	<4.77	[53]
	TC	WO_3-x_ QDs	MAA	EDGMA	–	PET, IFE	–	0.01–10.0 μmol/L 20.0–80.0 μmol/L	3.23 nmol/L and 6.37 μmol/L	Milk, egg	92.7–102.9	<1.59	[81]
		CDs	APTES	TEOS	Sol–gel method	ET	–	25–2000 nM	7.9 nM	Milk	94.2–103.7	1.5–5.3	[82]
		QDs	AM	EGDMA	Precipitation polymerization	ET	–	10–160 μmol/L	0.35 μmol/L	Milk	96.3–106.2	1.9–4.3	[83]
		CDs	AM	EGDMA	–	ET	Surface imprinting	0–50 nM	1.19 nM	River water, tap wate	99.13–102.75	0.67–0.98	[38]
	SDZ	CDs	4-VP	MBAAM	–	ET	–	2–20 μmol/L	4.2 nmol/L	Lake water, tap water	94.1–96.5	1.6–3.4	[84]
		CdTe QDs	AM	MBAAM	–	–	–	0–100 μmol/L	11 nmol/L	Tap water, lake water	92.0–95.1	<3.6	[85]
	FZD	CNDs	AMPS	EDGMA	Suspension polymerization	IFE	–	5–400 mg/L	1.98 mg/L	Chicken feed, honey, pork	90–110	<3.7	[39]
	CAP	QDs	APTES	TEOS	Sol–gel method	PET	Stimuli-responsive	0.015–0.12 nM 0.12–17.22 nM	0.009 nM	Lake water, chicken, fish, human urine	94.0–108.0	2.1–4.2	[68]
		NH2-UiO-66, CdTe QDs	APTES	TEOS	Sol–gel method	PET	–	10 pM–0.5 nM 0.5 nM–4.5 nM	3.8 pM	Meat, milk, honey	98.2–101.2	2.1–3.6	[86]
	CIP	CdTe QDs, CDs	APTES	TEOS	Sol–gel method	ET	–	0.5–20 μM	0.09 μM	Seawater	96.2–103.1	0.85–2.89	[87]
	TAP	CDs	APTES	TEOS	Sol–gel method	PET	–	5.0 nM–6.0 μM 6.0 μM–26.0 μM	50 μg/kg	Fish, shrimp, beef, milk	95.0–105.0	3.1–4.5	[88]
	PNG	CDs	APTES	TEOS	Sol–gel method	EF	–	1–32 nM	0.34 nM	Milk	98–103	<0.9	[89]
	CLP	CDs	APTES	TEOS	Sol–gel method	ET	_	0.1–3 μg/L	0.035 μg/L	Milk	96.5–106.6	1.1–3.5	[90]
	DMZ	CDs, CdSe	APTES	TEOS	Sol–gel method	–	–	0.06–14.1 μg/mL	47 pg/mL	Pork, honey, eggs	98.0–104.0	<3.9	[91]
	KS	QDs	APTES	TEOS	Sol–gel method	ET	–	3.00–105 μM	0.24 μM.	River water	102.5–112.6	1.12–3.85	[92]
	SS	QDs	APTES	TEOS	Sol–gel method	ET	–	3.00–118 μM	0.22 μM.	River water	97.7–106.6	2.17–6.21	[92]
	E2	CdTe QDs	APTES	TEOS	Sol–gel method	PET	Surface imprinting	0.011–50 μg/L	3.3 ng/L	Tap water, river water, seawater, milk	92.4–110.6	<2.5	[93]
	IMA	QDs	APTES	TEOS	Sol–gel method	PET	–	5.0–80.0 μM	1.4 μM	Puerariae Lobatae Radix, soil	85.5–98.0	<4.2	[94]
	DEL	CdSe/ZnS QDs	APTES	TEOS	_	_	–	0.01–40.0 mg/L	1.34 µg/L	Aquatic products, seawater	98.5–109.0	<7.5	[36]
	Mal	N-CDs, Eu-MOF	APTES	TEOS	Sol–gel method	–	–	1–10 μM	0.05 μM	Lettuce, tap water, soil	93.0–99.3	<3.1	[95]
	1-NP	QDs	APTES	TEOS	–	PET	–	6.0–140.0 μM	0.45 μM	Lycium barbarum, Dendrobium officinale	94–105	<4.41	[93]
	Propazine	QDS	APTES	TEOS	Sol–gel method	PET	–	0.005–2.000 mg/L	0.001 mg/L	Seawater, fish	92.0–114.6	<6.3	[96]
	2,4-D	CdTe QDs, NBD	4-VP	EGDMA	–	–	Surface imprinting	0−25 μM	0.13 μM	Drinking water, lake water, urban runoff water, paddy field water	99.2–100.8	1.5–3.3	[97]
		NBD	APTES	TEOS	Sol–gel method	PET	Surface imprinting	0.1–3 μM	0.023 μM	River water, tap water	94–108	<5	[32]
		CdTe QDs	4-VP	EGDMA	–	–	Surface imprinting	0–25 μM	0.12 μM	Milk	96.0–104.0	≤4.0	[98]
		CdTe QDs, NBD	APTES	TEOS	Sol–gel method	FRET	Surface imprinting	0.56–80 μM	90 nM	Soybean sprouts, lake water	86.2–109.5	<4.19	[78]
		CdTe QDs, NBD	4-VP	EGDMA	–	–	Surface imprinting	0−15 μM	0.13 μM	Milk	96.0−103.2	1.5−5.5	[99]
		CdTe QDs,	APTES	TEOS	–	ET	Surface imprinting	0.51–80 μmol/L	0.17 μmol/L	Cucumber	96.6–104.2	5.5–6.1	[100]
		CDs, FBM	APTES	TEOS	Sol–gel method	PET, IFE	–	1.0 × 10^−12^−1.2 × 10^−9^ M	7.5 × 10^−13^ M	River water, grass, cabbage, apple	7.1−103.3	1.3–2.7	[101]
	IDP	QDs	APTES	TEOS	–	ET	–	5 ng/mL–0.5 μg/mL	3.55 ng/mL	River water and corn	97.64–109.88	1.00–4.65	[73]
	TMX	CDs	APTES	TEOS	Sol–gel method	–	–	0.05–25 μM	13.5 nM	Fruit, Chinese cabbages, river water	91.40–105.7	1.64–3.49	[102]
	LC	CDs	APTES	TEOS	–	ET	Surface imprinting	1–150 μg/L	0.048 μg/L	Tap water, tea, cucumber, apple	87.93–101.4	1.5–5.1	[103]
	CF	SiO_2_@Y_2_O_3_:(Eu^3+^, Tb^3+^)	MAA	DMAEA	Sol–gel method	FRET, PET	_	10–100 μg/ml	4 μg/ml	Rhubarb, wolfberry	85.7–92.2	<3	[41]
	Pretilachlor	QDs	APTES	TEOS	Microemulsion polymerization	PET	Surface imprinting	0.001–5.0 mg/L	0.05 μg/L	Fish, river water	92.2–107.6	<6.5	[104]
	Difenoconazole	CDs, NBD	APTES	TEOS	Sol–gel method	PET	Surface imprinting	0.3~60 μmol/L	75 nmol/L	Tomato	102.1–111.2	3.1–4.2	[55]
		CDs, CdTe QDs	APTES	TEOS	–	IFE	–	–	0.18 μg/mL	Cucumber	102.2–108.8	4.7–5.6	[105]
	FA	CdTe QDs	APTES	TEOS	Sol–gel method	–	Surface imprinting	0.05–50 ppm	0.005 ppm	FA tablets, milk powder	89.39–103.43	<3.37	[76]
		QDs	APTES	TEOS	Sol–gel method	PET	–	0.01–50 ppm	0.0052 ppm	Milk powder, folic acid tablets, porcine serum	99.5–108.0	<3.0	[75]
		CdTe QDs,	APTES	TEOS	Sol–gel method	PET	Surface imprinting	0.23–113 μM	48 nM	Pinach, broccoli, tomatoes, oranges	94.8–104.2	–	[59]
	AAs	QDs	APTES	TEOS	Sol–gel method	PET	Multi-Template Imprinting	24–1200 nM	9.58 nM	Asarum	90.10–97.62	1.3–2.4	[106]
	ME	QDs	APTES	TEOS	Sol–gel method	IFE	Multi-Template Imprinting	17.1–343 nM	6.46 nM	Asarum	97.82–107.93	1.3–4.0	[106]
	AAI	CDs	4-VP	EGDMA	–	PET		1.0–120.0 μmol/L	0.45 μmol/L	Asarum	5.5–107.3	2.0	[107]
	MTX	CDs	APTES	TEOS	Copolymerization	IFE	–	5–2000 ng/mL	1.5 ng/mL	Rabbit plasma, parenteral	97.80–99.20	1.28–2.53	[108]
	DA	QDs, CDs	AM, VPBA	MBAAM	Precipitation polymerization	–	–	0–600 nM	12.35 nM	Human serum	98.02–104.06	<4.45	[109]
	DA	CdTe QDs	VPBA	MBAAm	–	ET	Surface imprinting	0–1.2 × 10^−6^ M	(100–150) × 10^−9^ M	Human serum	100.14–104.46	<5	[110]
	CTD	CDs	APTES	TEOS	Sol–gel method	ET	Surface imprinting	0.5–1000 nM	0.15 nM	Blood	96.12–107.40	2.87–3.96	[111]
	AA	CdTeS QDs, ZnCdS QDs	APTES	TEOS	Sol–gel method	ET	–	1–500 μM	0.78 μM	Vitamin C tablets	96.0–99.0	<1.7	[112]
		CuNCs, CDs	APTES	TEOS	Sol–gel method	–	Surface imprinting	4.0–22.0 μM	1.56 μM	Vitamin C tablet, human serum	81.69–106.11	0.10–0.44	[44]
	ACO	FeS_2_ QDs	MAA, AM	EDGMA	Sol–gel method	ET	–	0.05–5.0 μM	24 nM	Fuzi Lizhong Pills	95.2–103.1	<5.0	[66]
	Metronidazole	CDs	APTES	TEOS	Sol–gel method	–	–	25–1000 nmol/L	7.2 nmol/L	Honey	92.3–95.1	2.5–4.6	[113]
Biomarkers analysis	Histamine	QDs	APTES	TEOS	Sol–gel method	PET	–	1–60 μM	21.9 nM	Mackerel, frozen atlantic cod, canned tuna	96.52–105.32	1.57–5.57	[114]
	AD	BA-EuMOFs	–	TEOS	–	FRET	Surface imprinting	1–50 mg/L	0.26 mg/L	Human urine	96.11–101.79	<2.87	[115]
	BSA	QDs	APTES	TEOS	Sol–gel method	PET	–	2–64 μM	0.5 μM	Milk, power milk, human serum, bovine calf serum	95.9–104.8	<4	[16]
	L-Tyr	CdTe QDs	APTES	TEOS	Sol–gel method	IFE	Surface imprinting	1.0 × 10^–10^–2.5 × 10^−8^ M	8.0 × 10^−11^ M	Urine, serum	99.07–101.18	2.00–3.57	[17]
	dsDNA	QDs	APTES	TEOS	Sol–gel method	PRET	Surface imprinting	0.089–1.79 μg/mL	19.48 ng/mL	Urine	102.6–106.0	2.07–3.15	[26]
	CNP	NBD, CDs	APTES	TEOS	Sol–gel method	PET	Surface imprinting	5–80 pg/mL	2.87 pg/mL	Human serum	97.3–104	<4.7	[116]
	BHb	CdTe QDs	APTES	TEOS	Sol–gel method	PET	Surface imprinting	0.025−3 μM	7.8 nM	Urine	99.25−111.7	<3.2	[117]
	PE	CDs	APTES	TEOS	–	FRET	Surface imprinting	5–200 ng/mL	1.5 ng/mL	Human serum	95.94–104.26	1.62–4.65	[49]

SDZ: sulfadiazine, TAP: thiamphenicol, CLP: chloramphenicol, DMZ: dimenidazole, KS: kanamycin sulfate, SS: streptomycin sulphate, IMA: imazapyr, LC: λ-Cyhalothrin, DA: dopamine, ACO: aconitine, MAA: methacrylic acid, EGDMA: ethylene glycol dimethacrylate, APTES: 3-aminopropyltriethoxysilane, TEOS: tetraethyl orthosilicate, AM: acrylamide, MBAAM: N,N-methylbisacrylamide, 4-VP: 4-Vinylpyridine, DMAEA: dimethylaminoethyl methacrylate, VPBA: 4-vinylphenylboronic acid.

## Data Availability

Data are contained within the article.

## References

[1] Husain Khan A., Abdul Aziz H., Palaniandy P., Naushad M., Cevik E., Zahmatkesh S. (2023). Pharmaceutical Residues in the Ecosystem: Antibiotic Resistance, Health Impacts, and Removal Techniques. Chemosphere.

[2] El Agrebi N., Traynor K., Wilmart O., Tosi S., Leinartz L., Danneels E., de Graaf D.C., Saegerman C. (2020). Pesticide and Veterinary Drug Residues in Belgian Beeswax: Occurrence, Toxicity, and Risk to Honey Bees. Sci. Total Environ..

[3] Gosho M., Nagashima K., Sato Y. (2012). Study Designs and Statistical Analyses for Biomarker Research. Sensors.

[4] Kraus V.B. (2018). Biomarkers as Drug Development Tools: Discovery, Validation, Qualification and Use. Nat. Rev. Rheumatol.

[5] Kowalska M., Woźniak M., Kijek M., Mitrosz P., Szakiel J., Turek P. (2022). Management of Validation of HPLC Method for Determination of Acetylsalicylic Acid Impurities in A New Pharmaceutical Product. Sci. Rep..

[6] Orlandini S., Hancu G., Szabó Z.-I., Modroiu A., Papp L.-A., Gotti R., Furlanetto S. (2022). New Trends in the Quality Control of Enantiomeric Drugs: Quality by Design-Compliant Development of Chiral Capillary Electrophoresis Methods. Molecules.

[7] Saravanakumar K., Park S., Sathiyaseelan A., Kim K.-N., Cho S.-H., Mariadoss A.V.A., Wang M.-H. (2021). Metabolite Profiling of Methanolic Extract of Gardenia jaminoides by LC-MS/MS and GC-MS and Its Anti-Diabetic, and Anti-Oxidant Activities. Pharmaceuticals.

[8] Lionetto L., Ulivieri M., Capi M., De Bernardini D., Fazio F., Petrucca A., Pomes L.M., De Luca O., Gentile G., Casolla B. (2021). Increased Kynurenine-To-Tryptophan Ratio in the Serum of Patients Infected with SARS-CoV2: An Observational Cohort Study. BBA-Mol. Basis Dis..

[9] Wu C., Dougan T.J., Walt D.R. (2022). High-Throughput, High-Multiplex Digital Protein Detection with Attomolar Sensitivity. ACS Nano.

[10] Barik B., Mohapatra S. (2022). Selective Visual Detection of Histamine and Ascorbic Acid through the Rapid Gel-Sol Transition of Luminescent Alginate Hydrogel. Sens. Actuators B.

[11] Wang C., Liu M., Wang Z., Li S., Deng Y., He N. (2021). Point-Of-Care Diagnostics for Infectious Diseases: From Methods to Devices. Nano Today.

[12] Fernandes R.S., de Oliveira Silva J., Gomes K.B., Azevedo R.B., Townsend D.M., de Paula Sabino A., Branco de Barros A.L. (2022). Recent Advances in Point of Care Testing for COVID-19 Detection. Biomed. Pharmacother..

[13] You J., Liu H., Zhang R., Pan Q., Sun A., Zhang Z., Shi X. (2022). Development and Application of Tricolor Ratiometric Fluorescence Sensor Based on Molecularly Imprinted Nanoparticles for Visual Detection of Dibutyl Phthalate in Seawater and Fish Samples. Sci. Total Environ..

[14] Chen Y., Tang Y., Liu Y., Zhao F., Zeng B. (2022). Kill Two Birds with One Stone: Selective and Fast Removal and Sensitive Determination of Oxytetracycline Using Surface Molecularly Imprinted Polymer Based on Ionic Liquid and ATRP Polymerization. J. Hazard. Mater..

[15] Wang X., Yu S., Wang J., Yu J., Arabi M., Fu L., Li B., Li J., Chen L. (2020). Fluorescent Nanosensor Designing via Hybrid of Carbon Dots and Post-Imprinted Polymers for the Detection of Ovalbumin. Talanta.

[16] Wang L., Zhao L. (2022). A Novel Nanocomposite Optosensing Sensor Based on Porous Molecularly Imprinted Polymer and Dual Emission Quantum Dots for Visual and High Selective Detection of Bovine Serum Albumin. Colloids Surf. A.

[17] Tang S., Zhao P., Wu X., Chen Y., Tang K., Zhou S., Fu J., Lei H., Yang Z., Zhang Z. (2022). A Dual-Emission Ratiometric Fluorescence Capillary Imprinted Sensor Based on Metal-Organic Frameworks for Sensitive Detection of L-Tyrosine. Sens. Actuators B.

[18] Miao J., Ji W., Yu J., Cheng J., Huang Y., Arabi M., Zhou N., Li B., Zhang Z., Chen L. (2023). A Triple-Emission Ratiometric Fluorescence Sensor Based on Carbon Dots-Au Nanoclusters Nanocomposite for Detection of Tetracycline. Sens. Actuators B.

[19] Wang Y., Pan M., Yu X., Xu L. (2020). The Recent Advances of Fluorescent Sensors Based on Molecularly Imprinted Fluorescent Nanoparticles for Pharmaceutical Analysis. Curr. Med. Sci..

[20] Wen Y., Sun D., Yu J., Qi J., Zhang Z., Song Z., Wang X., Liu H., Chen L., Li J. (2023). Recent Advances in Molecular Imprinting-Based Ratiometric Fluorescence Sensors. Sci. Sin. Chim..

[21] Chen L., Wang X., Lu W., Wu X., Li J. (2016). Molecular Imprinting: Perspectives and Applications. Chem. Soc. Rev..

[22] Chen L., Xu S., Li J. (2011). Recent Advances In Molecular Imprinting Technology: Current Status, Challenges and Highlighted Applications. Chem. Soc. Rev..

[23] Lu H., Xu S. (2017). Visualizing BPA by Molecularly Imprinted Ratiometric Fluorescence Sensor Based on Dual Emission Nanoparticles. Biosens. Bioelectron..

[24] Li J., Sun D. (2022). Molecularly Imprinted Ratiometric Fluorescence Nanosensors. Langmuir.

[25] Lu H., Yu C., Xu S. (2019). A Dual Reference Ion-Imprinted Ratiometric Fluorescence Probe for Simultaneous Detection of Silver (I) and Lead (II). Sens. Actuators B.

[26] Arslan T., Guney O. (2020). Ratiometric Sensor Based on Imprinted Quantum Dots-Cationic Dye Nanohybrids for Selective Sensing of dsDNA. Anal. Biochem..

[27] Chen Y., Tang K., Zhou Q., Wang X., Zhang Z. (2023). Bimetallic Nanozyme Triple-Emission Fluorescence Intelligent Sensing Platform-Integrated Molecular Imprinting for Ultrasensitive Visual Detection of Triclosan. Spectrochim. Acta A Mol. Biomol. Spectrosc..

[28] Liu L., Ga L., Ai J. (2022). Ratiometric Fluorescence Sensing with Logical Operation: Theory, Design and Applications. Biosens. Bioelectron..

[29] Liu Z., Feng L., Hou J., Lv X., Ning J., Ge G., Wang K., Cui J., Yang L. (2014). A Ratiometric Fluorescent Sensor for Highly Selective Detection of Human Carboxylesterase 2 and Its Application in Living Cells. Sens. Actuators B.

[30] Peng M., Kaczmarek A.M., Van Hecke K. (2022). Ratiometric Thermometers Based on Rhodamine b and Fluorescein Dye-Incorporated (Nano) Cyclodextrin Metal-Organic Frameworks. ACS Appl. Mater. Interfaces.

[31] Wu L., Huang C., Emery B.P., Sedgwick A.C., Bull S.D., He X.P., Tian H., Yoon J., Sessler J.L., James T.D. (2020). Forster Resonance Energy Transfer (Fret)-Based Small-Molecule Sensors and Imaging Agents. Chem. Soc. Rev..

[32] Chu B., Yu Z., Meng L., Xu N. (2023). A Magnetic Molecular Imprinting-Based Fluorescence Probe for Sensitive and Selective Detection of 2,4-D Herbicide. Luminescence.

[33] Mohanta D., Chakraborty P. (2024). Nanoscale Matter and Principles for Sensing and Labeling Applications.

[34] Shao K., Guo L., Zhong Y., Zhang L., Lu Z., Wang D. (2024). Carbon Quantum Dots for Rapid and Ratiometric Fluorescence Determination of Hypochlorite. ACS Appl. Nano Mater..

[35] An Y., Bi A., Cheng Z., Du Y., Gao T., Grimm J., He S., Jiang X., Jokerst J.V., Kim T. (2020). Fluorescent Imaging in Medicinal Chemistry.

[36] You J., Hao G., Gan X., Chen R., Chen Y., Zhang Z., Sun A., Liu H., Shi X. (2024). Extreme Gradient Boosting-Enhanced Molecularly Imprinted Fluorescence Nanosensor for Rapid Identification and Visual Detection of Deltamethrin in Seawater and Aquatic Products. Sens. Actuators B.

[37] Zhu R., Lai M., Zhu M., Liang H., Zhou Q., Li R., Zhang W., Ye H. (2021). A Functional Ratio Fluorescence Sensor Platform Based on the Graphene/Mn-ZnS Quantum Dots Loaded with Molecularly Imprinted Polymer for Selective and Visual Detection Sinapic Acid. Spectrochim. Acta A Mol. Biomol. Spectrosc..

[38] Liu X., Wang T., Wang W., Zhou Z., Yan Y. (2019). A Tailored Molecular Imprinting Ratiometric Fluorescent Sensor Based on Red/Blue Carbon Dots for Ultrasensitive Tetracycline Detection. J. Ind. Eng. Chem..

[39] Zhang S., Mao Y., Song T., Zhao X., Song Z., Wang W. (2023). Ratiometric Fluorescence Probe Molecularly Imprinted Polymer Encapsulating N, S, B Doped Carbon Nanodots from Waste Clematis Chinensis Osbeck for Sensing Furazolidone. Carbon.

[40] Lv S., Zhang K., Zhu L., Tang D. (2020). Zif-8-Assisted Nayf4:Yb,Tm@Zno Converter with Exonuclease Iii-Powered Dna Walker for Near-Infrared Light Responsive Biosensor. Anal. Chem..

[41] Shen S., Long Z., Lu Y., Chen J. (2022). Fluorescence Detection of Carbofuran in Aqueous Extracts Based on Dual-Emission SiO_2_@Y_2_O_3_:(Eu^3+^,Tb^3+^)@MIP Core-Shell Structural Nanoparticles. Luminescence.

[42] Lu Y., Chen W. (2012). Sub-Nanometre Sized Metal Clusters: From Synthetic Challenges to the Unique Property Discoveries. Chem. Soc. Rev..

[43] Wang Z., Chen B., Rogach A.L. (2017). Synthesis, Optical Properties and Applications of Light-Emitting Copper Nanoclusters. Nanoscale Horiz..

[44] Pirot S.M., Omer K.M. (2022). Surface Imprinted Polymer on Dual Emitting MOF Functionalized with Blue Copper Nanoclusters and Yellow Carbon Dots as A Highly Specific Ratiometric Fluorescence Probe for Ascorbic Acid. Microchem. J..

[45] Li Y., Lu H., Xu S. (2024). The Construction of Dual-Emissive Ratiometric Fluorescent Probes Based on Fluorescent Nanoparticles for the Detection of Metal Ions and Small Molecules. Analyst.

[46] Förster T. (1984). Zwischenmolekulare Energiewanderung und Fluoreszenz. Ann. Der Phys..

[47] Chen G., Song F., Xiong X., Peng X. (2013). Fluorescent Nanosensors Based on Fluorescence Resonance Energy Transfer (Fret). Ind. Eng. Chem. Res..

[48] Li Z., Cui X., Xiao M., Miao J., Zhao B., Lin Z. (2021). An Fret-Ict-Based Ratiometric Fluorescent and Colorimetric Probe for pH Monitoring in Lysosomes and Water. Dye. Pigment..

[49] Fu Y., Jin H., Bu X., Gui R. (2019). Magnetic and Fluorescent Nanohybrids with Surface Imprinting Silica as A Dual-Functional Sensing Platform for Ratiometric Fluorescence Detection of Phycoerythrin. J. Mater. Chem. C.

[50] Chen S., Yu Y., Wang J. (2018). Inner Filter Effect-Based Fluorescent Sensing Systems: A Review. Anal. Chim. Acta.

[51] Zhang M., Cao X., Li H., Guan F., Guo J., Shen F., Luo Y., Sun C., Zhang L. (2012). Sensitive Fluorescent Detection of Melamine in Raw Milk Based on the Inner Filter Effect of Au Nanoparticles on the Fluorescence of CdTe Quantum Dots. Food Chem..

[52] Hu X., Guo Y., Zhang J., Wang X., Fang G., Wang S. (2022). A Signal-Amplified Ratiometric Fluorescence Biomimetic Sensor Based on the Synergistic Effect of IFE and AE for the Visual Smart Monitoring of Oxytetracycline. Chem. Eng. J..

[53] Li Y., Wu Y., Wang Y., Cao Y., Liu Y., Fang G., Wang S. (2024). Smartphone-Integrated Molecularly Imprinted Ratiometric Fluorescent Sensor for Selective and Visual Detection of Doxycycline in Lake Water and Foodstuff. ACS Sustain. Chem. Eng..

[54] Georgiev N.I., Dimitrova M.D., Todorova Y.D., Bojinov V.B. (2016). Synthesis, Chemosensing Properties and Logic Behaviour of a Novel Ratiometric 1,8-Naphthalimide Probe Based on ICT and PET. Dye. Pigment..

[55] Hao G., Chen L., Zhang Z., Ma X., Li J., Yang X. (2020). Environmentally Friendly Ratiometric Fluorescent Microfluidic Paper Chip for Rapid Detection of Difenoconazole. Sci. Sin. Chim..

[56] Miao X., Wu C., Li F., Zhang M. (2023). Fast and Visual Detection of Biogenic Amines and Food Freshness Based on ICT-Induced Ratiometric Fluorescent Probes. Adv. Funct. Mater..

[57] Zhang Z., Kang M., Tan H., Song N., Li M., Xiao P., Yan D., Zhang L., Wang D., Tang B.Z. (2022). The Fast-Growing Field of Photo-Driven Theranostics Based on Aggregation-Induced Emission. Chem. Soc. Rev..

[58] Huang C., Luo Y., Li J., Liu C., Zhou T., Deng J. (2021). pH-Regulated H_4_Tcpe@Eu/Amp Icp Sensor Array and Its Fingerprinting on Test Papers: Toward Point-Of-Use Systematic Analysis of Environmental Antibiotics. Anal. Chem..

[59] Li C., Yang Q., Wang X., Arabi M., Peng H., Li J., Xiong H., Chen L. (2020). Facile Approach to the Synthesis of Molecularly Imprinted Ratiometric Fluorescence Nanosensor for the Visual Detection of Folic Acid. Food Chem..

[60] Arabi M., Ostovan A., Li J., Wang X., Zhang Z., Choo J., Chen L. (2021). Molecular Imprinting: Green Perspectives and Strategies. Adv. Mater..

[61] Li J., Fu J., Yang Q., Wang L., Wang X., Chen L. (2018). Thermosensitive Molecularly Imprinted Core-Shell Cdte Quantum Dots as a Ratiometric Fluorescence Nanosensor for Phycocyanin Recognition and Detection in Seawater. Analyst.

[62] Chen R., Kang S., Li J., Lu L., Luo X., Wu L. (2021). Comparison and Recent Progress of Molecular Imprinting Technology and Dummy Template Molecular Imprinting Technology. Anal. Methods.

[63] Qi Z., Xiang C., Tian X., Xu X. (2022). Facile Synthesis of Molecularly Imprinted Ratiometric Fluorescence Sensor for Ciguatoxin p-Ctx-3c Detection in Fish. Foods.

[64] Li P., Fu H., Bai Z., Feng X., Qi J., Song X., Hu X., Chen L. (2023). A Dummy Molecularly Imprinted Ratiometric Fluorescence Nanosensor for the Sensitive Detection of Guanidyl-Microcystins in Environmental Water. Analyst.

[65] Shen R., Yu Y., Lan R. (2021). Progress in Application of Dual/Multi-Template Molecularly Imprinted Polymers. Chin. J. Anal. Chem..

[66] Luo K., Chen H., Zhou Q., Yan Z., Su Z., Li K. (2020). A Facile One Step Solvothermal Controllable Synthesis of FeS_2_ Quantum Dots with Multiple Color Emission for the Visual Detection of Aconitine. Spectrochim. Acta A Mol. Biomol. Spectrosc..

[67] Alaei H.S., Tehrani M.S., Husain S.W., Panahi H.A., Mehramizi A. (2018). Photo-Regulated Ultraselective Extraction of Azatioprine Using a Novel Photoresponsive Molecularly Imprinted Polymer Conjugated Hyperbranched Polymers Based Magnetic Nano-Particles. Polymer.

[68] Tang K., Chen Y., Wang X., Zhou Q., Lei H., Yang Z., Zhang Z. (2023). Smartphone-Integrated Tri-Color Fluorescence Sensing Platform Based on Acid-Sensitive Fluorescence Imprinted Polymers for Dual-Mode Visual Intelligent Detection of Ibuprofen, Chloramphenicol and Florfenicol. Anal. Chim. Acta.

[69] Gui R., Jin H., Bu X., Fu Y., Wang Z., Liu Q. (2019). Recent Advances in Dual-Emission Ratiometric Fluorescence Probes for Chemo/Biosensing and Bioimaging of Biomarkers. Coord. Chem. Rev..

[70] Lu H., Wei D., Zheng R., Xu S. (2019). Post-Imprinting Modification Based on Multilevel Mesoporous Silica for Highly Sensitive Molecularly Imprinted Fluorescent Sensors. Analyst.

[71] Huang X., Song J., Yung B.C., Huang X., Xiong Y., Chen X. (2018). Ratiometric Optical Nanoprobes Enable Accurate Molecular Detection and Imaging. Chem. Soc. Rev..

[72] Li H., Tian Y., Tan L., Wang N., Qiao Y., Wang J. (2024). A Double-Emission Molecularly Imprinted Ratiometric Fluorescent Sensor Based on Carbon Quantum Dots and Fluorescein Isothiocyanate for Visual Detection of p-Nitroaniline. Mikrochim. Acta.

[73] Wei Z., Zhang W., Wang S., Han Y., Feng D., Ma Y., Deng B., Chen Z., Mao J., Xu F. (2023). A Ratiometric Fluorescent Sensor Based on Molecularly Imprinted Multilevel Mesoporous Silica for Highly Sensitive Detection of Imidacloprid. Dye. Pigment..

[74] Cai Y., You J., You Z., Dong F., Du S., Zhang L. (2018). Profuse Color-Evolution-Based Fluorescent Test Paper Sensor for Rapid and Visual Monitoring of Endogenous Cu^2+^ in Human Urine. Biosens. Bioelectron..

[75] Yang Q., Li C., Li J., Wang X., Arabi M., Peng H., Xiong H., Chen L. (2020). Rational Construction of A Triple Emission Molecular Imprinting Sensor for Accurate Naked-Eye Detection of Folic Acid. Nanoscale.

[76] Wen Y., Sun D., Fu X., Jin Y., Yu J., Xu L., Song Z., Chen L., Li J. (2024). Molecular Imprinting-Based Ratiometric Fluorescence Nanosensor and Kit for Rapid and Visual Detection of Folic Acid. ACS Appl. Nano Mater..

[77] Fu J., Zhou S., Zhao P., Wu X., Tang S., Chen S., Yang Z., Zhang Z. (2022). A Dual-Response Ratiometric Fluorescence Imprinted Sensor Based on Metal-Organic Frameworks for Ultrasensitive Visual Detection of 4-Nitrophenol in Environments. Biosens. Bioelectron..

[78] Zhang Z., Ma X., Li B., Zhao J., Qi J., Hao G., Jianhui R., Yang X. (2020). Fluorescence Detection Of 2,4-Dichlorophenoxyacetic Acid by Ratiometric Fluorescence Imaging on Paper-Based Microfluidic Chips. Analyst.

[79] Hu B., Zhao W., Chen L., Liu Y., Ma Z., Yan Y., Meng M. (2024). Enhanced Molecularly Imprinted Fluorescent Test Strip for Rapid and Visual Detection of Norfloxacin via A Smartphone. Molecules.

[80] Miao J., Yu J., Zhao X., Chen X., Zhu C., Cao X., Huang Y., Li B., Wu Y., Chen L. (2024). Molecular Imprinting-Based Triple-Emission Ratiometric Fluorescence Sensor with Aggregation-Induced Emission Effect for Visual Detection of Doxycycline. J. Hazard. Mater..

[81] Zhou Y., Sha T., Liu D., Liao B., Li K. (2023). Molecularly Imprinted Ratiometric Fluorescence Detection of Tetracycline Based on Its Fluorescence Enhancement Effect Caused by Tungsten Trioxide Quantum Dots. Spectrochim. Acta A Mol. Biomol. Spectrosc..

[82] Sun X., Jiang M., Chen L., Niu N. (2021). Construction of Ratiometric Fluorescence MIPs Probe for Selective Detection of Tetracycline Based on Passion Fruit Peel Carbon Dots and Europium. Mikrochim. Acta.

[83] Wei X., Chen H. (2019). Ratiometric Fluorescence Molecularly Imprinted Sensor Based on Dual-Emission Quantum Dots Hybrid for Determination of Tetracycline. Anal. Bioanal. Chem..

[84] Yang W., Kuai M., Wu C., Yang W., Cao L., Zhang Y., Chen T., Cao Y., Wang B., Xu W. (2023). Dual-Emission Ratiometric Fluorescence Sensors Based on Silica, Molecularly Imprinted Polymers and Carbon Quantum Dots for High-Sensitivity and High-Selectivity Detection of Sulfadiazine. Polym. Int..

[85] Wu C., Zhang Y., Liu Y., Si H., Liu T., Yang W. (2022). Preparation and Recognition Performance of Ratio-Fluorescent Probe Based on Sulfadiazine Imprinted Polymers. Chin. J. Anal. Lab..

[86] Wu X., Tang S., Zhao P., Tang K., Chen Y., Fu J., Zhou S., Yang Z., Zhang Z. (2023). One-Pot Synthesis of Ternary-Emission Molecularly Imprinted Fluorescence Sensor Based on Metal-Organic Framework for Visual Detection of Chloramphenicol. Food Chem..

[87] Wang N., Majid A., Wang K., Tan L., Li H., Wang J. (2023). Composite Molecularly Imprinted Ratiometric Fluorescent Sensor Based on Carbon Dots and CdTe Quantum Dots for Visual Detection of Ciprofloxacin in Seawater. Mater. Sci. Semicond. Process.

[88] Fu J., Zhou S., Wu X., Tang S., Zhao P., Tang K., Chen Y., Yang Z., Zhang Z., Chen H. (2022). Down/Up-Conversion Dual-Mode Ratiometric Fluorescence Imprinted Sensor Embedded with Metal-Organic Frameworks for Dual-Channel Multi-Emission Multiplexed Visual Detection of Thiamphenicol. Environ. Pollut..

[89] Jalili R., Khataee A., Rashidi M.R., Razmjou A. (2020). Detection of Penicillin G Residues in Milk Based on Dual-Emission Carbon Dots and Molecularly Imprinted Polymers. Food Chem..

[90] Jalili R., Khataee A. (2020). Application of Molecularly Imprinted Polymers and Dual-Emission Carbon Dots Hybrid for Ratiometric Determination of Chloramphenicol in Milk. Food Chem. Toxicol..

[91] Su L., Li D., Du Y., Jiang G., Han S., Qin S. (2020). Preparation and Characterization of Molecularly Imprinted Ratio Fluorescent Probes for Dimenidazole. Chin. J. Anal. Lab..

[92] Ma Y., Jin X., Xing Y., Ni G., Peng J. (2019). Construction of An NAND Logic Gate Based on Molecularly Imprinted Dual-Emission Quantum Dot Composites for the Detection of Antibiotics. Anal. Methods.

[93] Wang N., Li H., Tian Y., Tan L., Cheng S., Wang J. (2024). Molecularly Imprinted Ratiometric Fluorescence Sensor for Visual Detection of 17beta-Estradiol In Milk: A Generalized Strategy Toward Imprinted Ratiometric Fluorescence Construction. Mikrochim. Acta.

[94] Zhou J., Xiao Y., Huang J., Huang M., Zhang S., Li K. (2024). A Novel Molecular Imprinted Probe with Hybrid Ratio Fluorescent for Visual Detection of Imazapyr. Microchem. J..

[95] Yang L., Hu W., Pei F., Liu Z., Wang J., Tong Z., Mu X., Du B., Xia M., Wang F. (2024). A Ratiometric Fluorescence Imprinted Sensor Based on N-CDs and Metal–Organic Frameworks for Visual Smart Detection of Malathion. Food Chem..

[96] Liu H., You J., Liu C., Zhang Z., Sun A., Hao G., Shi X. (2024). Machine Learning-Assisted Wide-Gamut Fluorescence Visual Test Paper for Propazine Determination in Fish and Seawater Samples. Sens. Actuators B.

[97] Cui Y., Li X., Wang X., Liu Y., Hu X., Chen S., Qu X. (2024). One-Pot Preparation of Ratiometric Fluorescent Molecularly Imprinted Polymer Nanosensor for Sensitive and Selective Detection of 2,4-Dichlorophenoxyacetic Acid. Sensors.

[98] Li Q., Zhang W., Liu X., Zhang H. (2022). Preparation of Complex Biological Sample-Compatible “Turn-On”-Type Ratiometric Fluorescent Molecularly Imprinted Polymer Microspheres via One-Pot Surface-Initiated ATRP. Mikrochim. Acta.

[99] Hou Y., Zou Y., Zhou Y., Zhang H. (2020). Biological Sample-Compatible Ratiometric Fluorescent Molecularly Imprinted Polymer Microspheres by RAFT Coupling Chemistry. Langmuir.

[100] Hao G., Zhang Z., Ma X., Zhang R., Qin X., Sun H., Yang X., Rong J. (2020). A Versatile Microfluidic Paper Chip Platform Based on MIPs for Rapid Ratiometric Sensing of Dual Fluorescence Signals. Microchem. J..

[101] Chen Y., Tang K., Zhou Q., Wang X., Wang R., Zhang Z. (2023). Integrating Intelligent Logic Gate Dual-Nanozyme Cascade Fluorescence Capillary Imprinted Sensors for Ultrasensitive Simultaneous Detection of 2,4-Dichlorophenoxyacetic Acid and 2,4-Dichlorophenol. Anal. Chem..

[102] Dai Y., Xu W., Hong J., Zheng Y., Fan H., Zhang J., Fei J., Zhu W., Hong J. (2023). A Molecularly Imprinted Ratiometric Fluorescence Sensor Based on Blue/Red Carbon Quantum Dots for the Visual Determination of Thiamethoxam. Biosens. Bioelectron..

[103] Zhu X., Han L., Liu H., Sun B. (2022). A Smartphone-Based Ratiometric Fluorescent Sensing System for On-Site Detection of Pyrethroids by Using Blue-Green Dual-Emission Carbon Dots. Food Chem..

[104] Liu C., Liao J., Zheng Y., Chen Y., Liu H., Shi X. (2022). Random Forest Algorithm-Enhanced Dual-Emission Molecularly Imprinted Fluorescence Sensing Method for Rapid Detection of Pretilachlor in Fish and Water Samples. J. Hazard. Mater..

[105] Hao G., Tian H., Zhang Z., Qin X., Yang T., Yuan L., Yang X. (2023). A Dual-Channel and Dual-Signal Microfluidic Paper Chip for Simultaneous Rapid Detection of Difenoconazole and Mancozeb. Microchem. J..

[106] Du Z., Li Y., Zeng C., Zhong Y., Wang S., Liu W., Chen Q., Pang M., Wang Y., Zhu R. (2024). Dual-Template Molecularly Imprinted Double Emission Proportional Fluorescence Sensor Based on CsPbBr3 and CsPb(Br/I)3 Perovskite Quantum Dots for Visual, Selective and Sensitive Detection of Methyl Eugenol and Aristolochic Acid A. Sens. Actuators B.

[107] Ye J., Cai X., Zhou Q., Yan Z., Li K. (2020). Molecularly Imprinted Ratiometric Fluorescent Probe for Visual and Fluorescent Determination of Aristolochic Acid I Based on a Schiff-Base Fluorescent Compound. Microchim. Acta.

[108] Alanazi A.Z., Alhazzani K., Mostafa A.M., Barker J., El-Wekil M.M., Ali A.B.H. (2024). Selective and Reliable Fluorometric Quantitation of Anti-Cancer Drug in Real Plasma Samples Using Nitrogen-Doped Carbon Dots after MMIPs Solid Phase Microextraction: Monitoring Methotrexate Plasma Level. J. Pharm. Biomed. Anal..

[109] Liu X., Fang Y., Zhu D., Wang J., Wu Y., Wang T., Wang Y. (2023). Hollow Structure Molecularly Imprinted Ratiometric Fluorescence Sensor for the Selective and Sensitive Detection of Dopamine. Analyst.

[110] Wang J., Dai J., Xu Y., Dai X., Zhang Y., Shi W., Sellergren B., Pan G. (2019). Molecularly Imprinted Fluorescent Test Strip for Direct, Rapid, and Visual Dopamine Detection in Tiny Amount of Biofluid. Small.

[111] Ling J., Zhang W., Cheng Z., Ding Y. (2022). High-Sensitivity Detection for Cantharidin by Ratiometric Fluorescent Sensor Based on Molecularly Imprinted Nanoparticles of Quantum Dots. J. Ind. Eng. Chem..

[112] Yang M., Wang C., Liu E., Hu X., Hao H., Fan J. (2021). A Novel Ascorbic Acid Ratiometric Fluorescent Sensor Based on ZnCdS Quantum Dots Embedded Molecularly Imprinted Polymer and Silica-Coated CdTeS Quantum Dots. J. Mol. Liq..

[113] Cheng X., Gao X., Qiao Z., Yu J., Fu Y., Chen L. (2020). Synthesis of Carbon Quantum Dot Molecularly Imprinted Composites and Detection of Metronidazole by Fluorescence Method. Chin. J. Anal. Lab..

[114] Wang T., Wang Y., Zeng Y., Tian X., Xu X. (2024). A Molecular Imprinted Ratiometric Fluorescence Sensor Based on Blue/Orange MXene Quantum Dots for Visual Detection of Histamine. Food Chem..

[115] Cheng P., Guo W., Li R., Yang Y., Du Q. (2023). Dual Recognition Ratio Fluorescence-Based Sensor for Sensitive Detection of Adenosine. Microchem. J..

[116] He H., Cao M., Hu J., Zhu L., Su C., Du S., Yang J., Tang Y., Chen L. (2020). Fluorescent Turn-On Assay of C-type Natriuretic Peptide Using A Molecularly Imprinted Ratiometric Fluorescent Probe with High Selectivity and Sensitivity. Mikrochim. Acta.

[117] Yang Q., Li J., Wang X., Xiong H., Chen L. (2019). Ternary Emission of a Blue-, Green-, and Red-Based Molecular Imprinting Fluorescence Sensor for the Multiplexed and Visual Detection of Bovine Hemoglobin. Anal. Chem..

[118] Farooq S., Wu H., Nie J., Ahmad S., Muhammad I., Zeeshan M., Khan R., Asim M. (2022). Application, Advancement and Green Aspects of Magnetic Molecularly Imprinted Polymers in Pesticide Residue Detection. Sci. Total Environ..

[119] Wang H., Xiao Y., Huang J., Huang M., Li K. (2024). A Molecularly Imprinted Ratiometric Fluorescent Sensor for Visual Detection of 1-Naphthol Based on Fluorescence-Enhanced CdTeS QDs via APTES Modification. Mikrochim. Acta.

[120] Yang L., Hu W., Pei F., Du B., Tong Z., Mu X., Xia M., Wang F., Liu B. (2024). Novel Dual-Emission Fluorescence Imprinted Sensor Based on Mg, N-CDs and Metal-Organic Frameworks for Rapid and Smart Detection of 2, 4, 6-Trinitrophenol. Talanta.

[121] Turnipseed S.B., Storey J.M., Wu I.L., Andersen W.C., Madson M.R. (2019). Extended Liquid Chromatography High Resolution Mass Spectrometry Screening Method for Veterinary Drug, Pesticide and Human Pharmaceutical Residues in Aquaculture Fish. Food Addit. Contam. Part. A Chem. Anal. Control Expo. Risk Assess..

[122] Ensafi A.A., Nasr-Esfahani P., Rezaei B. (2017). Simultaneous Detection of Folic Acid and Methotrexate By An Optical Sensor Based on Molecularly Imprinted Polymers on Dual-Color Cdte Quantum Dots. Anal. Chim. Acta.

[123] Fang C., Zhang X., Dong Z., Wang L., Megharaj M., Naidu R. (2018). Smartphone App-Based/Portable Sensor for the Detection of Fluoro-Surfactant PFOA. Chemosphere.

[124] Huang T., Xu Y., Meng M., Li C. (2022). Pvdf-Based Molecularly Imprinted Ratiometric Fluorescent Test Paper with Improved Visualization Effect for Catechol Monitoring. Microchem. J..

[125] Xu Y., Huang T., Hu B., Meng M., Yan Y. (2021). An Ultrasensitive Pvdf-Based Molecularly Imprinted Fluorescent Test Strip for the Rapid and Off-Line Detection of 4-Np With Improved Anti-Coffee Ring Effect. J. Mater. Chem. C.

[126] Wang L., Wang X., Cheng L., Ding S., Wang G., Choo J., Chen L. (2021). Sers-Based Test Strips: Principles, Designs and Applications. Biosens. Bioelectron..

[127] Dittrich P.S., Manz A. (2006). Lab-On-a-Chip: Microfluidics in Drug Discovery. Nat. Rev. Drug Discov..

[128] Nishat S., Jafry A.T., Martinez A.W., Awan F.R. (2021). Paper-Based Microfluidics: Simplified Fabrication and Assay Methods. Sens. Actuators B.

[129] Zhang J., Wang W., Shao J., Chen J., Dong X. (2024). Small Molecular Cyanine Dyes for Phototheranostics. Coord. Chem. Rev..

[130] Zhang Z., Chasteen J.L., Smith B.D. (2024). Cy5 Dye Cassettes Exhibit Through-Bond Energy Transfer and Enable Ratiometric Fluorescence Sensing. J. Org. Chem..

[131] Udhayakumari D. (2024). Mechanistic Innovations in Fluorescent Chemosensors for Detecting Toxic Ions: PET, ICT, ESIPT, FRET and AIE Approaches. J. Fluoresc..

[132] Lu X., He W. (2021). Research Advances in Excited State Intramolecular Proton Transfer Fluorescent Probes Based on Combined Fluorescence Mechanism. Chin. J. Anal. Chem..

[133] Chi H., Liu G. (2023). A Novel Dual-Template Molecularly Imprinted Polymer Ratiometric Fluorescence Sensor Based on Three-Emission Carbon Quantum Dots for Accurate Naked-Eye Detection of Aflatoxin B1 and Zearalenone in Vegetable Oil. Microchem. J..

[134] Gao Q., Li X., Ning G., Leng K., Tian B., Liu C., Tang W., Xu H., Loh K.P. (2018). Highly Photoluminescent Two-Dimensional Imine-Based Covalent Organic Frameworks for Chemical Sensing. Chem. Commun..

[135] Guo L., Yang L., Li M., Kuang L., Song Y., Wang L. (2021). Covalent Organic Frameworks for Fluorescent Sensing: Recent Developments and Future Challenges. Coord. Chem. Rev..

[136] Gao X., Lu W., Wang Y., Song X., Wang C., Kirlikovali K.O., Li P. (2022). Recent Advancements of Photo- And Electro-Active Hydrogen-Bonded Organic Frameworks. Sci. China Chem..

[137] Mao C., Dai R., Zhao L. (2024). Research on Photoelectrochemical Sensing Applications of Hydrogen- Bonded Organic Frameworks. J. Mol. Struct..

